# The tRNA-modifying function of MnmE is controlled by post-hydrolysis steps of its GTPase cycle

**DOI:** 10.1093/nar/gkt320

**Published:** 2013-04-27

**Authors:** Silvia Prado, Magda Villarroya, Milagros Medina, M.-Eugenia Armengod

**Affiliations:** ^1^RNA Modification and Mitochondrial Diseases Laboratory, Centro de Investigación Príncipe Felipe, 46012-Valencia, Spain, ^2^Departamento de Bioquímica, Biología Molecular y Celular. Instituto de Biocomputación y Física de Sistemas Complejos (BIFI). Universidad de Zaragoza, 50009-Zaragoza, Spain and ^3^Biomedical Research Networking Centre in Rare Diseases (CIBERER) (node U721), Spain

## Abstract

MnmE is a homodimeric multi-domain GTPase involved in tRNA modification. This protein differs from Ras-like GTPases in its low affinity for guanine nucleotides and mechanism of activation, which occurs by a *cis*, nucleotide- and potassium-dependent dimerization of its G-domains. Moreover, MnmE requires GTP hydrolysis to be functionally active. However, how GTP hydrolysis drives tRNA modification and how the MnmE GTPase cycle is regulated remains unresolved. Here, the kinetics of the MnmE GTPase cycle was studied under single-turnover conditions using stopped- and quench-flow techniques. We found that the G-domain dissociation is the rate-limiting step of the overall reaction. Mutational analysis and fast kinetics assays revealed that GTP hydrolysis, G-domain dissociation and P_i_ release can be uncoupled and that G-domain dissociation is directly responsible for the ‘ON’ state of MnmE. Thus, MnmE provides a new paradigm of how the ON/OFF cycling of GTPases may regulate a cellular process. We also demonstrate that the MnmE GTPase cycle is negatively controlled by the reaction products GDP and P_i_. This feedback mechanism may prevent inefficacious GTP hydrolysis *in vivo*. We propose a biological model whereby a conformational change triggered by tRNA binding is required to remove product inhibition and initiate a new GTPase/tRNA-modification cycle.

## INTRODUCTION

GTP-binding proteins (G proteins or GTPases) regulate a multitude of cellular processes, including protein biosynthesis and translocation, ribosome assembly, signal transduction, membrane trafficking and cell cycle control (1). The common property shared among these proteins is the presence of a structural module, the G-domain, in which conserved residues (motifs G1–G4) are invariably involved in binding of guanine nucleotides and Mg^2+^, hydrolysis of GTP or control of conformational changes associated with the binding and hydrolysis of GTP. These changes are primarily confined to two highly flexible regions called switches I and II (which include motifs G2 and G3, respectively) and are crucial for the function of all G proteins. The GTPase cycle is regulated by the intrinsic properties of each G protein, as well as by the specific factors with which the G protein interacts.

The GTPase cycle of Ras-like proteins has been widely investigated and often is used as the reference model (1,2). This cycle requires participation of GTPase-activating proteins (GAPs) and guanine nucleotide-exchange factors (GEFs) because Ras-like proteins have a low intrinsic hydrolase activity and a high affinity for guanine nucleotides. RasGAPs stimulate hydrolysis by supplying a catalytic arginine (the arginine finger) into the active site. This residue stabilizes an endogenous glutamine lying adjacent to motif G3, which in turn stabilizes the water molecule for the in-line attack, while the arginine neutralizes the negative charge of β- and γ-phosphate oxygens in the transition state. Ras-related proteins are in an active state when GTP bound; the GTP binding causes a conformational change in switches I and II that allows interaction with an effector. Hydrolysis of GTP leads to relaxation of switches I and II and to the inactive GDP-bound state of the protein. Usually, binding of effector and GAP are mutually exclusive events so that the effector has to be released before GAP interaction. Therefore, the biological and GTPase activities are independent one from another. After GTP hydrolysis, the protein may be again switched ‘ON’ by the exchange of bound GDP to GTP, mediated by GEFs.

Many G proteins, however, do not follow the prototypical Ras–GTPase cycle because their biochemical properties and activation mechanisms make the participation of GEFs and GAPs unnecessary (1–7).

MnmE (formerly TrmE) is a homodimeric multi-domain GTPase, conserved between bacteria and eukarya, which participates in and regulates a tRNA-modification pathway (8). MnmE has been classified as a HAS GTPase because the catalytic glutamine of Ras proteins is substituted by a hydrophobic amino acid (9). In spite of this, MnmE and its isolated G-domain exhibit a high intrinsic GTPase activity (10). Because of this property and the low affinity of MnmE for guanine nucleotides, its GTPase cycle proceeds efficiently *in vitro* without GAPs and GEFs (10–15). The MnmE GTPase activity is stimulated by a *cis*, nucleotide- and potassium-dependent dimerization of its G-domains (13). Thus, MnmE has been classified as both a G protein activated by nucleotide-dependent dimerization (GAD) and a cation-dependent (CD) GTPase (1–3). GADs typically show a reciprocal complementation of their active sites when dimerized, which allows the GTPase machinery to achieve the catalytically competent conformation.

**Figure 1.**
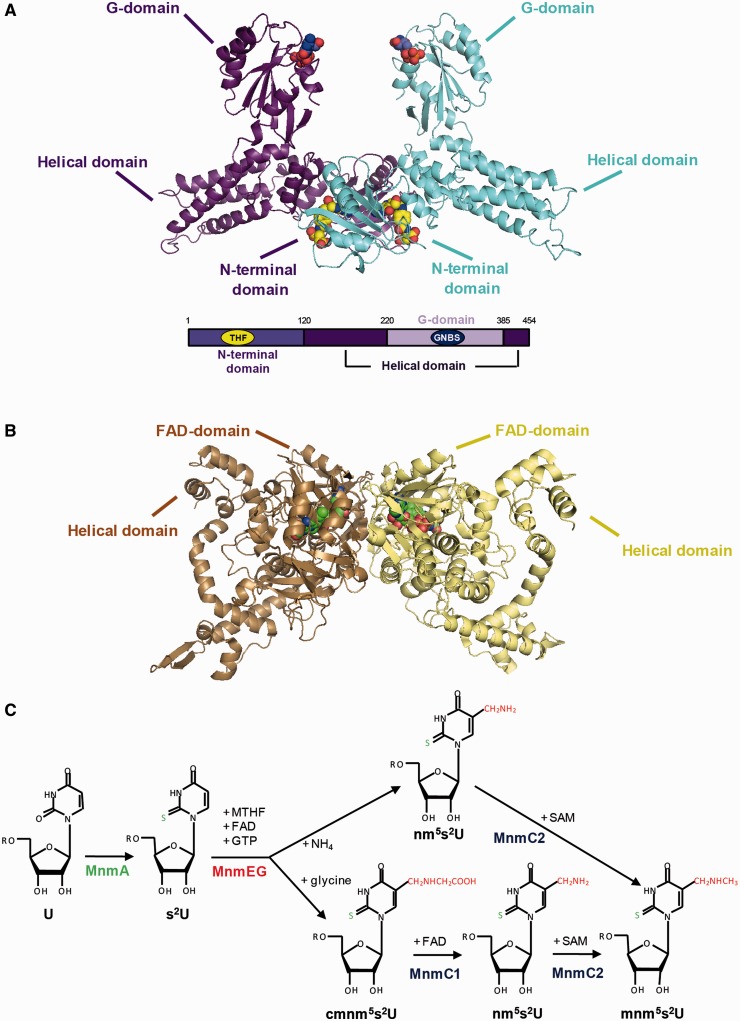
3D-structures of MnmE and GidA proteins. (**A**) Top: Model of the dimeric MnmE protein (blue/purple) shown in cartoon representation. GDP and 5-formyl-THF are shown in spheres coloured in CPK with carbon atoms in blue and yellow, respectively. The model was obtained by superimposition of two monomeric complete molecules MnmE·GDP·5-formyl-THF from *Chlorobium tepidum* (pdb 3GEE) (16) on the partial structure of the MnmE·5-formyl-THF dimer from *Thermotoga maritima* (pdb 1XZQ), where only the N-terminal domain B, but not the helical and G-domain of molecule B, were present in the crystal. Bottom: Domain composition of MnmE. (**B**) Cartoon representation of the *Aquifex aeolicus* MnmG dimer (brown/pale yellow) with FAD (pdb 2ZXI) (17). The FAD cofactor is represented in spheres coloured in CPK with carbon atoms in green. (**C**) Schematic of the MnmE-dependent modification pathway (8). MnmA carries out the thiolation at position 2 of the wobble uridine (U34), whereas the MnmEG complex catalyses the first step of the modification at position 5, which may occur through two different reactions that produce nm^5^U or cmnm^5^U. The bifunctional enzyme MnmC catalyses the last two steps in the biosynthesis of mnm^5^s^2^U by means of its FAD-dependent deacetylase and SAM-dependent methylase activities (MnmC1 and MnmC2, respectively). Abbreviations: s^2^, nm^5^s^2^U, cmnm^5^s^2^U and mnm^5^s^2^U mean 2-thiouridine, 5-aminomethyl-2-thiouridine, 5-carboxymethylaminomethyl-2-thiouridine and 5-methylaminomethyl-2-thiouridine, respectively. GNBS, THF, MTHF and SAM mean guanine nucleotide-binding site, tetrahydropholate, methylene-tetrahydrofolate and S-adenosyl-l-methionine, respectively.

Crystal structures of MnmE from several bacteria show a dimeric protein with each monomer (50 kDa) consisting of three domains: an N-terminal α/β domain responsible for constitutive dimerization and binding of tetrahydrofolate (THF); a central helical domain formed by residues from the middle and the C-terminal regions; and a G-domain, embedded within the helical domain, that conserves the canonical Ras-like fold (Figure 1A) (11,16). The X-ray structures show MnmE as a constitutive homodimer in which the highly mobile G-domains face each other. The MnmE G-domains dimerize in *cis* via their switch regions in the presence of potassium and GTP (or GDP and aluminium fluoride, AlF_x_, which mimics the γ-phosphate in the transition state) (13,16). Dimerization stabilizes the switch regions and reorients the catalytic residue (E282), which in turn positions a water molecule for the nucleophilic attack of the γ-phosphate group (13). The potassium ion, which is coordinated by the nucleotide and residues of switch I (the K-loop) and the P-loop (motif G1), plays a crucial role in the stabilization of the transition state. The ion provides a positive charge into the catalytic site in an analogous position to the arginine finger in the Ras–RasGAP system, thus contributing to neutralizing the negative charges in the transition state.

MnmE, together with the conserved FAD-binding protein MnmG (formerly GidA; Figure 1B), is involved in the modification of the wobble uridine of tRNAs decoding NNA/G codons belonging to split codon boxes (8,19–21). In *Escherichia coli*, the lack of the MnmE/MnmG-promoted modification causes translational errors, reduced growth rate, glucose-inhibited division, synthetic lethality and extreme sensitivity to acidic pH (10,12,19,22–26). The eukaryotic homologues of MnmE and MnmG are targeted to mitochondria and have been related to the pathogenesis of certain mitochondrial diseases (27–30). Recently, mutations in the human MnmG homologue have been shown to cause hypertrophic cardiomyopathy and lactic acidosis (31).

The *E. coli* MnmE and MnmG proteins form a functional α2β2 heterotetrameric complex (MnmEG) in which both proteins are interdependent (8,20,21). The MnmEG complex catalyses two different GTP- and FAD-dependent reactions on tRNA, which produce 5-aminomethyluridine and 5-carboxymethylaminomethyluridine in the wobble position by using ammonium and glycine, respectively, as substrates, and methylene-THF as the source behind the C5-methylene moiety formation (Figure 1C). In contrast to Ras-like proteins, MnmE must hydrolyse GTP for carrying out its biological function (12,18,24). However, the precise role of GTP hydrolysis in the tRNA modification remains unknown. Indeed, according to current models (8), the modification reaction itself does not need GTP hydrolysis. Given that the MnmE G-domain is relatively far from the active centre of the MnmEG complex (where methylene-THF and FAD are located), it is thought that the conformational changes associated with GTP hydrolysis are transmitted from the G-domain to both the remaining domains of MnmE and its partner MnmG, promoting structural rearrangements in the complex that are crucial for tRNA modification. Because dimerization of the G-domains is accompanied by large domain movements from an open to a closed state, it has been hypothesized that G-domain dimerization during GTP hydrolysis is required for orchestration of the tRNA-modification reaction (16,18). However, data from our group have suggested that a post-hydrolysis step could be involved in the functional activation of MnmE (12). Thus, the relationships between the GTPase cycle and the tRNA-modifying function of MnmE are still not fully understood. Additionally, if this protein does not require assistance of GEFs and GAPs, how is it regulated to prevent futile GTP consumption?

Therefore, our study addressed two main objectives: (i) to determine the timing of individual steps of the GTPase cycle and identify which one is directly responsible for the functional activation of MnmE; and (ii) to elucidate the regulatory mechanism that controls the ‘OFF’ state of MnmE.

Our data demonstrate that the MnmE GTPase cycle is a multi-step process in which the G-domain dissociation step is slower than the preceding GTP hydrolysis step and acts as the limiting step of the overall reaction rate. Mutational analysis indicates that GTP hydrolysis, G-domain dissociation and inorganic phosphate (P_i_) release can be uncoupled and supports the idea that conformational changes linked with G-domain dissociation are responsible for the functionally active state of MnmE. Moreover, we show that the GTPase cycle is negatively controlled by the reaction products GDP and P_i_. This control of the cycle may be a way to avoid futile GTP hydrolysis, regulating MnmE GTPase activity within the MnmEG complex.

## MATERIALS AND METHODS

### Bacterial strains, plasmids, growth conditions and general protein techniques

These procedures are described in the Supplementary Information. Strains and plasmids are listed in Supplementary Table S2.

### GTPase activity under multi-turnover conditions and measurement of *K*_D_ for mGTPγS

Hydrolysis of GTP and GTPγS was measured by a malachite green colorimetric assay for free P_i_, as previously described (24). Dissociation constants under equilibrium conditions were determined by titration of the proteins against fluorescent mant nucleotides (12).

### Single-turnover kinetics

Single-turnover kinetics of the MnmE GTPase cycle was initially performed by rapid mixing of MnmE (0.5–50 μM) with 5 μM of mGTP in a BioLogic SFM-300 with a MOS-450 optics stopped-flow apparatus. The mant fluorophore was excited at 360 nm and the change in fluorescence monitored through a 400 nm cut-off filter. Experiments were carried out at 20°C in 50 mM Tris pH 7.5, 5 mM MgCl_2_ and 150 mM KCl. Rapid mixing of mGTP and MnmE solutions at 1:1 proportion resulted in an absorbance increase, which took place within 0.5 s, and was fitted to a biphasic kinetic (GTP binding and G-domain dimerization), and a later decrease (0.5–17 s) that was fitted to a monophasic kinetic (G-domain dissociation). Time courses were fitted by means of the Biokine software (BioLogic, France). These experiments allowed us to obtain the rate constant at each MnmE concentration. By plotting the rate versus concentration to the hyperbolic equation *k*_obs_ = *k*_max_ × [MnmE] / (*K*_D_ + [MnmE]), we derived the maximum rate and the dissociation constant for each step. Thus, the maximum rate and the *K*_D_ for mGTP binding were 3896 ± 59 min^−1^ and 0.60 ± 0.03 μM, respectively. Maximum rates for dimerization and dissociation of G-domains were 869 ± 36 and 14.5 ± 0.9 min^−1^, respectively. We found that concentrations of MnmE above 5 µM (initial concentration) had no significant effect on the rates, and that rates close to the maximum were obtained by mixing solutions of MnmE and mGTP at 5 µM (final concentration in the mix: 2.5 μM). Similar results were obtained for the MnmE variants E282A and R256A. These experiments allowed us to select appropriate enzyme and substrate concentrations at which the enzyme is operating at maximum (or near maximum) rate in each step of the GTPase cycle. Accordingly, to analyse GTP binding, G-domain dimerization, and G-domain dissociation of MnmE and its variants, solutions at 5 μM of mGTP and protein were mixed 1:1 in the stopped-flow apparatus, and the change in fluorescence was monitored as indicated above. Experiments were carried out at 20°C in 50 mM Tris pH 7.5, 5 mM MgCl_2_ and 150 mM NaCl or KCl. A control experiment using the wild-type MnmE protein and GTPγS was carried out under the same conditions. To determine the single-turnover rate of GTP hydrolysis, a total of 100 μM of protein was mixed in a quench-flow apparatus with 100 μM of GTP in buffer A at 20°C. The experiment was stopped at various time points during 12 s with 1 M perchloric acid as quencher and neutralized with 8 M potassium acetate. Precipitated protein and salt were removed by centrifugation (5 min, 13 000*g*), and a volume of 100 μl of the supernatant was applied to a hydrophobic C18-column [ultra performance liquid chromatography (UPLC)] with 10 mM ammonium phosphate and 10 mM triethanolamine as the mobile phase. The percentage of uncleaved GTP was plotted as a function of time and fitted to one-phase exponential decay (GraphPad).

### Fast kinetic analysis of the dissociation of MnmE–mGDP complexes by FRET

MnmE (at 4 µM) was rapidly mixed with 4 μM of mGTP (initial concentrations) in the stopped-flow instrument and the increase of fluorescence detected during 500 s. After 1 min, unlabelled competitor GTP was added at different concentrations (0.8–800 μM). We monitored the fluorescence resonance energy transfer (FRET) from tryptophans located at the MnmE G-domain to the mant group of the bound GDP (excitation at 290 nm and emission at 400 nm). Data were collected at 10-ms intervals and curves fitted to a single-exponential function with the Biokine software.

### Dissociation rate of inorganic phosphate

P_i_ release from MnmE after GTP hydrolysis was followed by the fluorescence change of the MDCC (*N*-[2-(1-maleimidyl)ethyl]-7-(diethylamino)-coumarin-3-carboxamide)-labelled phosphate-binding protein (PBP) (32) in buffer A at 20°C by using a spectrophotometer (LS 50 B spectrophotometer, PerkinElmer Life Sciences) and the stopped-flow instrument. The first 7 s of the P_i_ release from the wild-type, T250S and R256A MnmE proteins measured by the spectrophotometer could not be registered yet the single-exponential fit provided us this information, which was corroborated by the stopped-flow measurements. Kinetics slower than those observed in T250S were completely registered by the spectrophotometer. Single-turnover P_i_ release was measured after mixing protein and GTP (1:1) in the presence of MDCC-PBP. The reaction mixtures contained purine nucleoside phosphorylase (0.1 U/ml) and 7-methylguanosine (200 μM) serving as a ‘P_i_ mop’ to take up trace amounts of contaminating P_i_ (32). Fluorescence of MDCC-PBP was excited at 425 nm and measured at 471 nm. The relative fluorescence was plotted as a function of time considering the first 15 s of the reaction and fitted to a single-exponential step (GraphPad).

### Inhibition assays

A solution of MnmE prepared with increasing GDP or P_i_ concentrations was mixed in a 1:1 proportion with different solutions of mGTP or GTP in the stopped-flow or quench-flow apparatus. All experiments were carried out in buffer A at 20°C, and kinetics of the various steps of the GTPase cycle were determined as specified above. The type of inhibition displayed by GDP and P_i_ and the inhibition constants were determined following the inhibitory effect on the G-domain dissociation step. Initial rate constants were determined by fitting the kinetic traces to single exponentials. Inhibition constants (*K*_IE_ and *K*_IES_*)* were calculated by using Dixon and [GTP] / v_o_ versus [P_i_] plots, respectively (33). IC_50_ for GDP was calculated using IC_50_ = *K*_IE_ (1 + [GTP_ce__l__lular_] / *K*_m_), assuming *K*_m_ = 710 μM for the wild-type MnmE. IC_50_ for P_i_ was calculated using IC_50_ = *K*_IE_ / (*K*_m_ / [GTP_cel__l__ular_]) + (*K*_IE_ / *K*_IES_). In this case, IC_50_ was assumed to be similar to the *K*_IES_ value in the wild-type and variant proteins given that the quotient (*K*_m_ / [GTP_ce__l__lular_]) is similar to (*K*_IE_ / *K*_IES_). The *K*_m_ values for T250S and G285A variants were 494 and 789 μM, respectively.

### Analysis of tRNA modification

Strains expressing MnmE proteins were grown until late log phase in LB broth with thymine. Then, cells were lysed, and tRNA was isolated and treated as described (21). tRNA hydrolysates were analysed by high-performance liquid chromatography (HPLC) (Develosil C30 column) or UPLC (Acquity UPLC BEH C18 column). The percentage of tRNA modification activity was calculated from the mnm^5^s^2^U/s^4^U ratio of absorbances at 314 nm.

### Statistical analysis

Values are expressed as the mean of a minimum of three independent experiments with a standard deviation, unless otherwise specified. In the case of the stopped-flow determinations, the fluorescence change in each experiment was averaged from at least six individual reactions and was best fitted to a double-exponential equation (mGTP binding and G-domain dimerization) or a single-exponential equation (G-domain dissociation) to determine the observed rate constants. The data from a minimum of three independent stopped-flow experiments are presented as the mean value (SD).

## RESULTS

### G-domain dissociation is the rate-limiting step of the MnmE GTPase cycle

**Figure 2.**
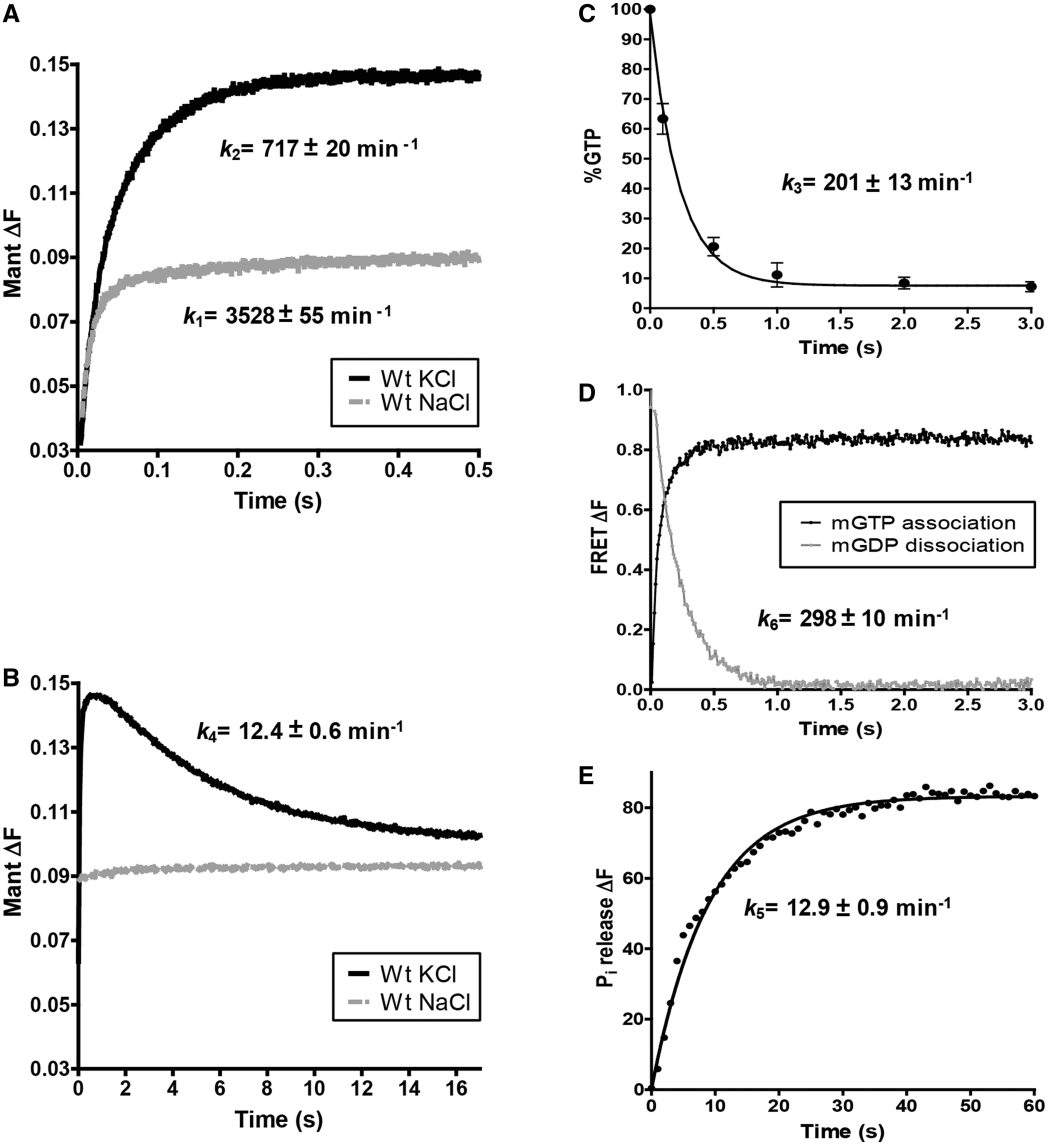
Fast kinetic analysis of the MnmE GTPase cycle. (**A** and **B**) Representative stopped-flow kinetic plots of mGTP binding and G-domain dimerization during the first 0.5 s, in (A), and G-domain dissociation, during 17 s, in (B). (**C**) GTP hydrolysis assessed by quench-flow and UPLC analysis. The averages of three independent experiments are shown. Error bars represent standard deviation. (**D**) Representative time course of mGDP dissociation from MnmE determined by stopped-flow FRET. MnmE was mixed with mGTP, and FRET was followed in real time (black line). Addition of an excess of unlabelled GTP promoted dissociation of the MnmE-bound mGDP resulting from GTP hydrolysis (grey line). (**E**) Representative time course of P_i_ release from MnmE after GTP hydrolysis fitted to a single-exponential equation (solid line). MnmE (10 μM) was mixed with 10 μM of GTP in the presence of MDCC-PBP protein (8.5 μM) in the stopped-flow instrument. All experiments were performed in the presence of KCl except controls where KCl was substituted by NaCl (A and B). Note that all indicated rate constants have been determined from at least three independent experiments.

The GTPase cycle of MnmE is thought to be a multi-step process with progression controlled by some step(s) after GTP binding. This suggestion was proposed because the *K*_m_ for GTP hydrolysis (determined by the release of P_i_ under steady-state conditions) is about 500-fold greater than the *K*_D_ for GTP analogues (*K*_m,_ 754 μM; *K*_D_, 1.51 μM) (12). Of interest, the *K*_m_ was reported to be only about 10-fold higher than the *K_D_* value when GTP hydrolysis was followed by GDP production from denatured samples (*K*_m_, 12 μM) (18). In both cases, the GTP turnover number (*k*_cat_) was similar (about 10 min^−1^). These results suggest that the P_i_ release could be a limiting step in the GTPase cycle and that it is delayed in relation to the GTP hydrolysis step. To test this hypothesis and to determine the nature and timing of individual steps of the MnmE GTPase cycle, we performed fast kinetic experiments (Figure 2) under single-turnover conditions.

In a first set of experiments, we monitored GTP binding, G-domain dimerization and G-domain dissociation by mant fluorescence with 2′-/3′-*O*-(*N*′-methylanthraniloyl) (m) GTP and stopped-flow spectrophotometry (Figure 2A and B). Rapid mixing of MnmE with mGTP led to an initial increase in fluorescence that followed a biphasic kinetics (the first 0.5 s; Figure 2A), and a later decrease that was fitted to a monophasic kinetics (0.5–17 s; Figure 2B). The first stretch of the fluorescence increase reached ∼0.095 relative fluorescence with a rate constant of *k*_1_ = 3527.9 min^−1^ (Figure 2A) and resulted from the binding of the mGTP to the protein. The second stretch, with an amplitude change from ∼0.095 to 0.15 relative fluorescence and a rate constant of *k*_2_ = 717 min^−1^ (Figure 2A), was due to the G-domain dimerization. This second stretch was not observed when MnmE bound to mGTP in a buffer containing NaCl in place of KCl, an expected result, given that G-domain dimerization, but not GTP binding, requires potassium for stabilization (13). Then, the fluorescence decay, from ∼0.15 to 0.10 relative fluorescence with a rate constant of *k*_4_ = 12.4 min^−1^, resulted from dissociation of the G-domains as a consequence of the GTP hydrolysis (Figure 2B). This decay reached a level of fluorescence (around 0.10 relative fluorescence) similar to that exhibited by the mGTP-bound protein, suggesting that GTP hydrolysis by MnmE led to dissociation of G domains but not to mGDP dissociation under our experimental conditions. As a control, we monitored nucleotide binding, G-domain dimerization and G-domain dissociation by mant fluorescence with mGTPγS, a slowly hydrolysable GTP analogue with a *k*_cat_ of 0.8 ± 0.1 min^−^^1^ (our own data). The kinetics of binding (*k*_1_ = 3476 min^−1^) and G-domain dimerization (*k*_2_ = 241 min^−1^) were similar and about 3-fold slower, respectively, than those observed with mGTP, whereas the kinetics of the fluorescence decay was 20-fold slower (*k*_4_ = 0.7 min^−1^), so that a decrease of only ∼15% in fluorescence was observed after 200 s from the beginning of the reaction (Supplementary Figure S1). These results support the previously stated notion that the fluorescence decay is a consequence of hydrolysis and G-domain dissociation (13).

To be sure that the fluorescence decay really monitors GTP hydrolysis as previously reported (13), we analysed, in a second set of experiments, the single-turnover GTPase reaction mediated by the full MnmE protein by quench-flow and subsequent UPLC analysis of the uncleaved GTP from the quenched samples (Figure 2C). To our surprise, the reaction showed a rate constant of 201 min^−1^, almost 20-fold faster than the rate constant of the fluorescence decay observed with the stopped-flow method (12.4 min^−1^; Figure 2, compare B and C). These results differ from those of a previous study (13) using a truncated MnmE protein lacking the N-terminal domain (ΔN-MnmE), which found that the GTP hydrolysis rate constant determined by quench-flow was in line with the fluorescence data, yet the G-domain dissociation kinetic was not calculated.

**Figure 3.**
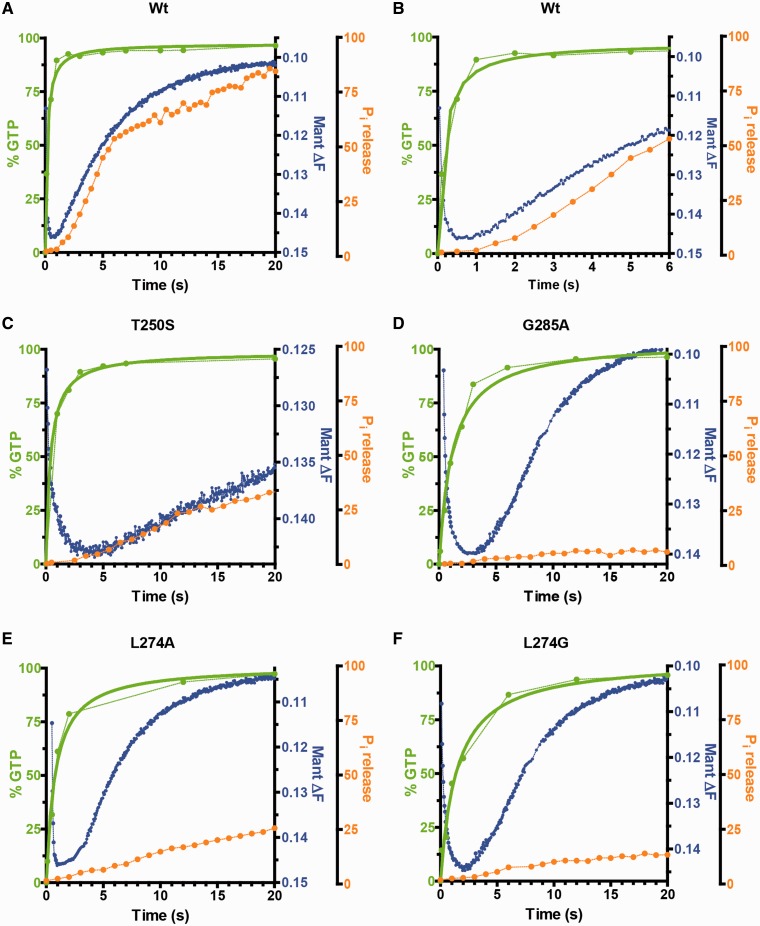
Comparison of kinetics of wt and variant MnmE proteins. Kinetics of GTP hydrolysis (green line), G-domain dissociation (blue line) and P_i_ release (orange line) of MnmE (**A** in 20 s and **B** in 6 s) and MnmE variants T250S, G285A, L274A and L274G (**C–F**, during 20 s of reaction) are shown. Data have been taken from Figures 2 (wt MnmE) and 7 (MnmE variants).

To explore whether the N-terminal domain of MnmE may affect the GTPase cycle, we compared the rate constants of GTP binding, G-domain dimerization, GTP hydrolysis and G-domain dissociation exhibited by three MnmE constructs: the isolated MnmE G-domain, the ΔN-MnmE protein and the MnmE full protein (referred to here as MnmE) (Table 1). The latter showed the slowest rate constant in each step of the GTPase cycle, with G-domain dissociation being the most affected (50% reduction in the rate constant with respect to the isolated G-domain). These data indicate that the N-terminal dimerization domain plays a significant and unexpected role in the GTPase cycle progression mainly by modulating the post-hydrolysis steps. Note, however, that the three MnmE constructs exhibited G-domain dissociation rates slower than the respective hydrolysis rates, suggesting that both steps are independent. In fact, a careful examination of the dissociation kinetics at short times (Figure 3A and B) reveals a delay of about 0.6 s in the dissociation step, as visualized by the section of the dimerization/dissociation curve running parallel to the *x*-axis (from 0.5 to 1.1 s; Figure 3B). This lag phase could include the time required for GTP hydrolysis, which is less than 1 s (Figure 3B, green line). If so, GTP hydrolysis and formation of a GDP•P_i_ intermediate do not involve a significant instantaneous conformational rearrangement at the G-domain active centre because there is no fluorescence change in the lag phase. Accordingly, the fluorescence decay observed in the stopped-flow experiments would be exclusively the result of the dissociation of the dimer.

**Table 1.** Fast kinetic parameters of different MnmE constructs

MnmE construct	GTP binding (*k*_1_)	G-domain dimerization (*k*_2_)	GTP hydrolysis (*k*_3_)	G-domain dissociation (*k*_4_)
Isolated G-domain	3956 ± 294 (100%)	825 ± 16 (100%)	301 ± 5 (100%)	24 ± 3 (100%)
ΔN-MnmE	3832 ± 143 (97%)	804 ± 12 (97%)	244 ± 26 (81%)	22 ± 2 (89%)
MnmE	3528 ± 55 (90%)	717 ± 20 (87%)	201 ± 13 (67%)	12 ± 1 (51%)

Each construct was mixed with mGTP in the stopped-flow instrument, and the mant fluorescence emission was followed in real time. GTP hydrolysis rate was determined from quench-flow experiments. Values are mean ± SD of a minimum of three independent experiments. Rate constant values expressed as a percentage of those calculated for the isolated G-domain are indicated between brackets. *k* values are given in min^−1^.

Finally, in a third set of experiments, we analysed the release of the reaction products GDP and P_i_. To study the GDP release rate, we used FRET from two neighbour tryptophan residues of the MnmE G-domain, both located at the GTP/GDP-binding site (W290 and W317; pdb 2GJA). After the mixing of MnmE with mGTP and the subsequent rapid mGTP hydrolysis, no mGDP dissociation was observed (Figure 2D, black line), thus confirming that this reaction product remains bound to MnmE under our experimental conditions (GDP concentration after GTP hydrolysis is 10-fold higher than the *K*_D_ value for GDP, which has been calculated to be around 0.5 µM) (11,13). Only the addition (when GTP hydrolysis was thought to be finished, 1 min after reaction initiation) of an excess (800 μM) of non-labelled competitor GTP promoted the mGDP release with a rate constant of about 300 min^−1^ (Figure 2D, grey line). The addition of competitor GTP at concentrations lower than 800 μM (from 0.8 to 500 μM) reduced the amplitude of the fluorescence change (so that mGDP was then released from a smaller number of MnmE molecules) but did not affect the rate constant (Supplementary Figure S2). In *E. coli* cells, the GTP and GDP concentrations are thought to be about 5 and 0.7 mM, respectively (34). Considering the similar affinity of MnmE for GDP and GTP (*K*_D_ ∼1 μM) (11–13; Supplementary Table S1) and the high GTP hydrolysis and GDP-exchange rates exhibited by the protein, the guanine nucleotide occupancy of MnmE *in vivo* may be dictated by the GTP–GDP pool, unless the exchange of guanine nucleotides is suppressed by an inhibitor factor or structural determinants.

**Figure 4.**
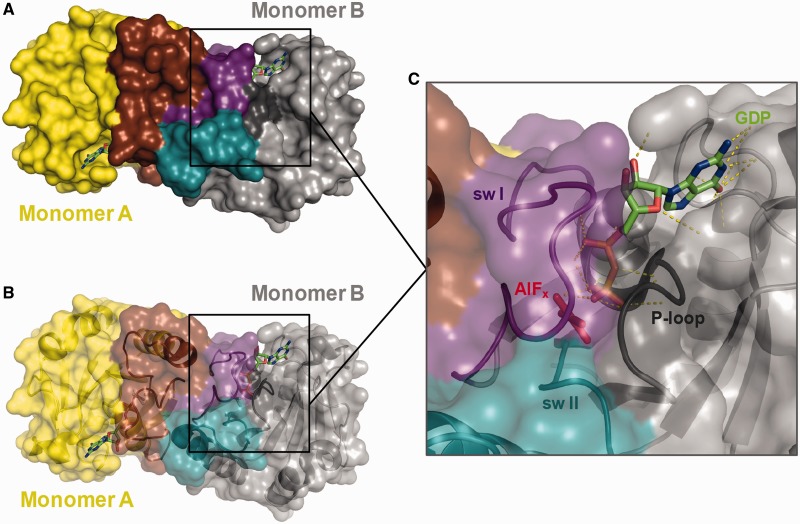
The MnmE G-domain dimerization builds a dead-end tunnel for GDP–AlF_x_. Dimeric structure of the MnmE G-domain in the presence of GDP–AlF_x_ and potassium (pdb 2GJA) represented in surface (**A**) and ribbon with semi-transparent surface (**B**). Monomer A is shown in yellow with switches I and II in brown, and monomer B is shown in grey with switches I and II in violet and blue, respectively. GDP and AlF_x_ are represented in sticks. (**C**) Detailed view showing the dead-end tunnel of monomer B formed by residues of the P-loop (black), the K-loop of switch I (violet) and switch II (blue). AlF_x_ is shown in red while GDP is coloured in CPK (with carbon atoms in green, oxygen atoms in red, phosphate atoms in orange and nitrogen atoms in blue). Dashed lines in yellow show the hydrogen bonds formed between GDP and the protein residues.

The release of P_i_ from MnmE was monitored by the fluorescence of MDCC-labelled PBP, which strongly increases upon binding of P_i_ (32). The rate of P_i_ release was 12.9 min^−1^ (Figure 2E). Given that in our assay the rate of P_i_ release depends on the rates of the previous steps, it appears that the P_i_ release follows G-domain dissociation. In fact, the structure of the G-domain dimer in the presence of GDP–AlF_x_ and potassium (pdb 2GJA) reveals that GDP–AlF_x_ is located at a dead-end tunnel where only the guanine of the GDP is exposed to the solvent, whereas the α and β phosphates and the AlF_x_ (which mimics the γ phosphate) are located in a deeper position (Figure 4). According to this structure, the tunnel is formed by residues of the P-loop, residues of the K-loop from switch I and residues of switch II such as G273, R275 or E282, which are directly involved in catalysis (13). Therefore, the dimer conformation, by stabilizing the switches in the K^+^-, GTP-bound conformation, prevents the release of not only P_i_ but also GDP (Figure 4). Consequently, we conclude that G-domain dissociation precedes P_i_ and GDP release. In this way, dissociation appears as the critical step that modulates the final rate of the MnmE GTPase cycle.

### GDP- and P_i_-binding negatively regulates the MnmE GTPase cycle

A question to be resolved is how the GTPase cycle of MnmE is controlled *in vivo* to prevent futile GTP consumption. Here, we tested the possibility that the MnmE GTPase activity is negatively regulated by the reaction products. To this end, the effects of GDP and P_i_ on the single-turnover GTPase reaction were measured by stopped- and quenched-flow assays as above. Data are shown in Table 2 and Supplementary Figure S3. We found that the rate and amplitude of the fluorescence enhancement associated with the GTP binding were reduced in the presence of GDP. Thus, the rate constant of GTP binding decreases by about 98% at 100 µM of GDP. A similar effect of GDP was observed on the remaining steps of the GTPase cycle, probably attributable to the primary influence on GTP binding. In contrast, P_i_ had a minor effect on GTP binding (the rate constant decreased by about 8% at 10 mM of P_i_) whereas it had greater effects on G-domain dimerization and GTP hydrolysis (40 and 76% decrease of the rate constant at 10 mM of P_i_, respectively).

**Table 2.** Inhibitory effect of GDP and P_i_ on the MnmE GTPase cycle

[GDP] or [P_i_]	GTP binding (*k*_1_)	G-domain dimerization (*k*_2_)	GTP hydrolysis (*k*_3_)	G-domain dissociation (*k*_4_)
[GDP] μM				
0	3322 ± 55	723 ± 20	201 ± 13	11.1 ± 0.6
10	645 ± 46	274 ± 26	49 ± 1	6.0 ± 0.5
50	207 ± 3	94 ± 3	25 ± 3	3.2 ± 0.3
80	90 ± 5	22 ± 2	12 ± 2	0.8 ± 0.1
100	45 ± 3 (98%)	10 ± 2 (98%)	8 ± 1 (96%)	0.6 ± 0.04 (95%)
[P_i_] mM				
0	3322 ± 55	723 ± 20	201 ± 13	11.1 ± 0.6
0.5	3213 ± 85	702 ± 11	123 ± 6	10.4 ± 0.3
2	3292 ± 23	603 ± 23	71 ± 7	7.4 ± 0.6
5	3128 ± 209	546 ± 50	55 ± 3	5.9 ± 0.8
10	3078 ± 127 (8%)	435 ± 32 (40%)	48 ± 1 (76%)	4.2 ± 0.4 (62%)

To determine the effect on GTP binding and G-domain dimerization and dissociation, a solution of MnmE (5 µM) containing increasing concentrations of GDP (0–100 µM) or P_i_ (0–10 mM) was mixed (1:1) with an mGTP solution (5 µM) in the stopped-flow apparatus, and the mant fluorescence emission was followed in real time. To determine the effect on GTP hydrolysis, a solution of MnmE (100 µM) containing different concentrations of GDP (0–100 µM) or P_i_ (0–10 mM) was mixed (1:1) with a solution of GTP (100 µM) in the quench-flow instrument. Results are mean ± SD of at least three independent experiments. Values in brackets indicate the percentage of inhibition at the indicated concentrations. *k* values are given in min^−1^.

Surprisingly, P_i_ showed a lower effect on G-domain dissociation than on GTP hydrolysis (62 versus 76% decrease of the rate constant at 10 mM of P_i_, respectively; Table 2 and Supplementary Figure S3B). It is possible that the inhibition of GTP hydrolysis by P_i_ results in dissociation of GTP-containing dimers, which produces an ‘atypical’ contribution to the fluorescence decrease because it is not related to GTP hydrolysis. Consequently, the inhibitory effect of P_i_ on G-domain dissociation falsely appears smaller than expected (see Supplementary Figure S3B). It should be pointed out that the effect of P_i_ on the G-domain dimers cannot be attributed to the high ionic strength produced by the higher P_i_ concentrations because the use of KCl at 150 mM in the reaction buffer had no effect on the rate constants of the GTPase cycle (Supplementary Figure S4). Altogether our data suggest that the major effect of P_i_ is on stabilization of the transition state.

**Figure 5.**
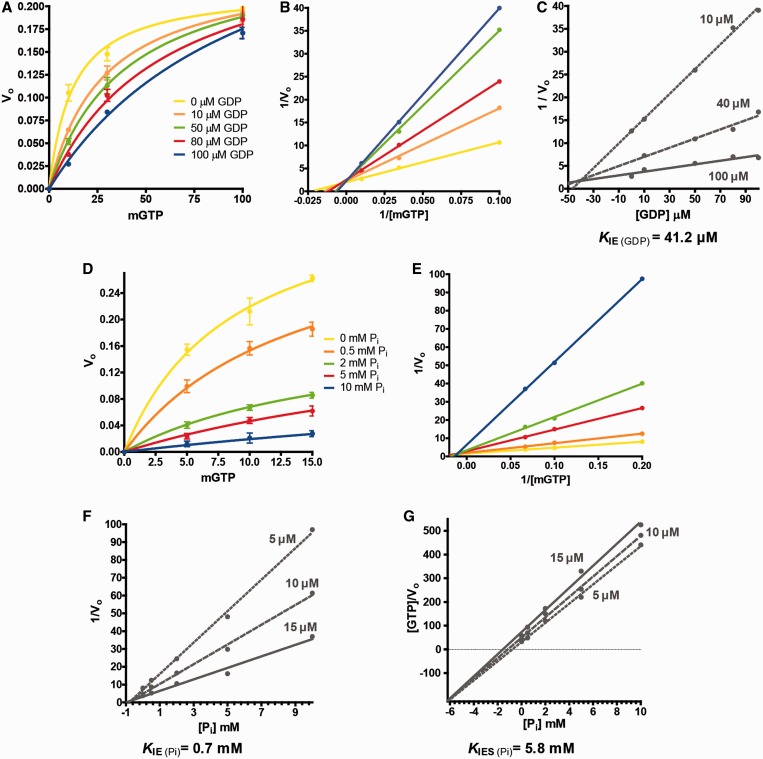
Determination of the inhibition constants for GDP and P_i_. (**A–C**) Effect of GDP on G-domain dissociation. A solution of MnmE (60 µM) containing different GDP concentrations (0–100 μM) was rapidly mixed in a 1:1 proportion with different solutions of mGTP (at 10, 40 or 100 μM) in a stopped-flow apparatus. Michaelis-Menten, double reciprocal and Dixon plots are shown in panels (A), (B) and (C), respectively. (**D–G**) Effect of P_i_ on G-domain dissociation. A solution of MnmE (5 μM) containing different P_i_ concentrations (0–10 mM) was mixed in a 1:1 proportion with mGTP (at 5, 10 or 15 μM) in the stopped-flow instrument. Michaelis-Menten (D), double reciprocal (E) and Dixon (F) plots are shown together with a [GTP] / V_o_ versus [P_i_] representation (G). The intersection point of lines in the Dixon plot provides the inhibition constant (*K*_IE_) of the enzyme for the inhibitor, while the intersection point in the [GTP] / V_o_ versus [P_i_] representation provides the inhibition constant of the enzyme-substrate complex (*K*_IES_) for the inhibitor. Each time point is an average from at least three independent experiments. As the data used in panels (A–C) and (D–E) were the same, the error bars (standard deviation) are only shown in panels (A) and (D).

To determine the type of inhibition produced by GDP and P_i_, we analysed the effect of increasing concentrations of GDP and P_i_ on the catalytic parameters of the G-domain dissociation (Figure 5A, B, D and E, and Table 3). Our data indicated that GDP interacts with MnmE in a competitive manner, increasing the *K_m_* value of GTP for MnmE from 19.5 μM in the absence of GDP to 77.6 μM in the presence of 100 μM of GDP, whereas *k_cat_* remained almost unchanged (Table 3). The inhibition constant (*K*_IE_) for GDP was determined to be 41.2 µM by using the Dixon plot (Figure 5C). This value, together with the *K*_m_ of the reaction (determined by the release of P_i_ under steady-state conditions; *K*_m_ = 710 µM), and the intracellular concentration of the substrate (GTP, 5 mM) (34), was used to calculate the GDP concentration responsible for 50% of maximal effect of inhibition (IC_50_), which was estimated to be 0.47 mM. This outcome suggests that about 75% of the MnmE molecules might be inhibited at the GDP intracellular concentration (0.7 mM) (34).

**Table 3.** Effect of GDP and P_i_ on *k_cat_* and *K*_m_ of G-domain dissociation

[GDP] or [P_i_]	*k_cat_*	*K*_m_
[GDP] μM		
0	0.23 ± 0.02	19.5 ± 0.7
10	0.27 ± 0.03	38.8 ± 1.8
50	0.30 ± 0.03	57.4 ± 3.7
80	0.32 ± 0.03	65.6 ± 5.7
100	0.35 ± 0.03	77.6 ± 4.3
[P_i_] mM		
0	0.41 ± 0.04	8.7 ± 0.8
0.5	0.37 ± 0.03	14.3 ± 0.5
2	0.19 ± 0.02	17.8 ± 1.2
5	0.20 ± 0.02	34.3 ± 2.4
10	0.14 ± 0.01	60.8 ± 3.3

Experiments were performed as indicated in the legend to Figure 5. The mean values ± SD were obtained from the Michaelis–Menten plots shown in Figure 5 (panels A and D). *k*_cat_ and *K*_m_ values are given in min^−1^ and μM, respectively.

The addition of P_i_ exhibited a non-competitive mixed-type inhibition pattern, given that it increased the *K_m_* value from 8.7 in the absence of P_i_ to 60.8 μM in the presence of 10 mM of P_i_, whereas the *k_cat_* value decreased from 0.41 min^−1^ to 0.14 min^−1^ at the same P_i_ concentrations (Table 3). The two inhibition constants, towards the enzyme (*K*_IE_) and towards the enzyme–substrate complex (*K*_IES_), were 0.67 mM and 5.8 mM, respectively (Figure 5F and G). These values reflect the higher affinity between the inhibitor and the free enzyme (*K*_IE_) compared with that between the inhibitor and the enzyme–substrate complex (*K*_IES_). Given that the *K*_IES_ value correlates with IC_50_ in this type of inhibition, our data suggest that almost 90% of the MnmE–GTP complexes might be inhibited at the physiological concentration of P_i_ (10 mM) (35). Therefore, we conclude that the GTP hydrolysis reaction catalysed by MnmE is negatively regulated by the final products GDP and P_i_.

### Dissecting the post-hydrolysis steps of the GTPase cycle by mutational analysis

**Figure 6.**
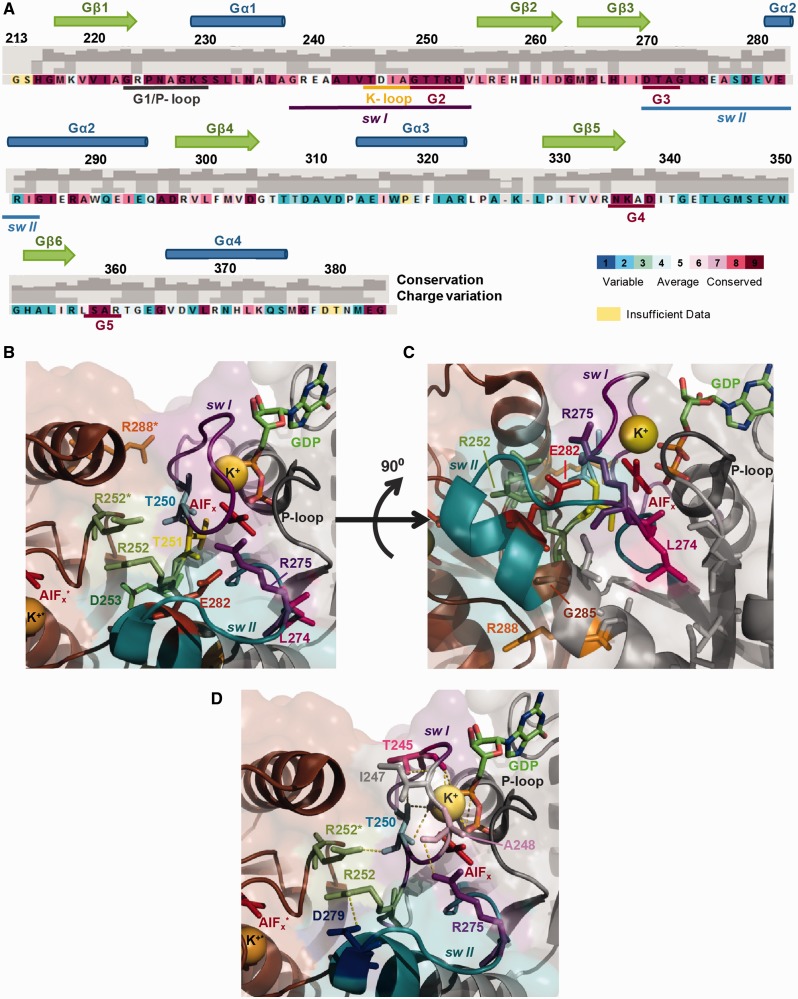
Residues of MnmE G-domain substituted in this study. (**A**) Secondary structure assignment as deduced from the GDP–AlF_x_ structure (pdb 2GJA) and sequence conservation of the MnmE G-domain. The degree of amino acid conservation and charge variation, which were calculated with ConSeq (37) using 150 sequences, are shown by coloured and grey bars in the corresponding files. (**B**) Catalytic centre of dimeric MnmE G-domain in the transition state mimic (pdb 2GJA). (**C**) Rotation of 90° of view (B) where the retracted conformation of L274 can be observed. (**D**) Ionic interactions of T250 residue in the MnmE wt protein. Residues are shown in sticks while the hydrogen bonds are indicated by yellow dashed lines. Residues and factors (K^+^ and AlF_x_) of the second protomer are indicated by an asterisk.

To obtain information about how GTP hydrolysis becomes the driving force of the tRNA modification function, we decided to use variant proteins presumably affected in different post-hydrolysis steps of the GTPase cycle. In a previous study on the role of residues located in or close to switches I and II of the MnmE G-domain, our group found no correlation between the catalytic activity and the biological effect of some *mnmE* mutations (12) (for updating data, see Table 4; for the location of specific residues on the sequence and on the structure of MnmE, see Figure 6). MnmE proteins carrying the amino acid substitutions T250S, R256A and R288A exhibited a similar catalytic activity (*k*_cat_ around 5–6 min^−1^ versus ∼11 min^−1^ for the wild-type protein), but only R256A produced wild-type phenotypic traits, such as those related to growth rate and translational accuracy (12). These results suggested that GTP hydrolysis is not sufficient for MnmE to promote tRNA modification (and, therefore, a wild-type phenotype), and that the deleterious substitutions could affect post-hydrolysis steps that play crucial roles in the MnmE function. Other replacements affecting the catalytic activity to a greater extent (*k*_cat_ around 1.5 min^−^^1^; Table 4), such as R252A, D253A and R275A, also differed among each other in their phenotypes (12). Therefore, kinetic analysis of all these proteins under single-turnover conditions may provide information about the step affected by a given substitution and its role in the functional activation of MnmE.

**Table 4.** GTP hydrolysis and tRNA modification activity of MnmE proteins

MnmE protein	Multiple-round GTP hydrolysis^a^	tRNA-modification activity^b^	
	*k_cat_*	mnm^5^s^2^U/s^4^U^c^	Activity (%)
Wt	11.39 ± 0.58	0.037	100
T250S	**5.63 ± 0.30**	**0.007**	**19**
T251A	0.24 ± 0.06	0	0
R252A	2.05 ± 0.23	0.002	5
D253A	1.34 ± 0.11	0	0
R256A	**5.28 ± 0.35**	**0.034**	**97**
L274G	**5.44 ± 0.11**	**0**	**0**
L274A	**6.71 ± 0.32**	**0**	**0**
L274Q	3.63 ± 0.20	0.007	19
R275A	**1.45 ± 0.27**	**0.018**	**51**
E282A	0.30 ± 0.02	0	0
E282D	8.57 ± 0.54	0.036	100
G285A	3.57 ± 0.16	0	0
G285I	0.78 ± 0.06	0	0
R288A	**5.26 ± 0.37**	**0.004**	**11**

Bold numbers are used to highlight the lack of correlation between the tRNA modification status and the GTPase activity in some representative cases.

^a^For multiple-turnover analysis, 2 μM of protein was incubated with increasing concentrations of GTP (0–2 mM), and hydrolysis was measured by the malachite green assay. Results are mean ± SD of at least three independent experiments. *k_cat_* values are given in min^−1^.

^b^tRNA from strains expressing the different MnmE proteins (wild-type or variants) was purified and degraded to nucleosides for HPLC/UPLC analysis. Nucleosides were monitored at 314 nm to maximize the detection of thiolated nucleosides. mnm^5^s^2^U (the final product of the MnmE-dependent pathway) and s^4^U (a nucleoside independent of the MnmE pathway and used herein as a reference) were identified by their UV spectra (36) and appropriate controls.

^c^The numbers were calculated as the absorbance of mnm^5^s^2^U relative to the absorbance of s^4^U at 314 nm. Each value is the mean of at least three independent experiments. Standard deviations were within ±10%, except for R252A and R288A where SD was around ±30%.

We also included in the ongoing study substitutions in the conserved residues L274 and G285, both located at switch II (Figure 6A–C). Structural analysis has indicated that switch II undergoes a remarkable rearrangement in the transition state whereby helix Gα2 is elongated by two additional turns and tilted by ∼33 degrees compared with the apo form (13). This rearrangement reorients the catalytic E282, which is stabilized by R275 (Figure 6B). The strictly conserved G285 is located at the second turn of the elongated Gα2 helix (Figure 6A and C), and we anticipated that its substitution by a different residue could perturb rearrangements at the catalytic centre during the GTPase cycle (Figure 6C). The hydrophobic residue L274, located at switch II but outside helix Gα2 (Figure 6A–C), occupies the same sequence position as the catalytic Gln in Ras-like GTPases. In MnmE, as in other HAS GTPases, this hydrophobic residue adopts a ‘retracted conformation’ in the transition state mimic, where it is positioned away from the GTP, thus avoiding interference with catalysis (Figure 6C) (9). The retracted conformation creates a space that is occupied by the catalytic residue E282. Leu274 is substituted by Ile or Val in other MnmE family members, so we hypothesized that a change of this residue to Ala, Gly or Gln could affect the catalytic machinery. Finally, substitutions in the catalytic residues E282 and T251 (12,13) were used as controls in our experiments.

First of all, we compared the catalytic activity of the new variants (L274G, L274A, L274Q, G285A and G285I) with the old ones and determined the tRNA-modifying capability of all variants (Table 4 and Supplementary Figure S5). Of interest, L274G and L274A exhibited a catalytic constant similar to that of R256A, but unlike this variant, L274G and L274A could not modify tRNA *in vivo*. The *k*_cat_ value for protein L274Q was smaller than for L274A and L274G. Paradoxically, the tRNA modification level was greater in L274Q than in L274A and L274G. The catalytic activity of G285A was similar to that of L274Q, but unlike this protein, G285A was totally deficient in tRNA modification. The change G285I drastically impaired MnmE GTPase activity and, accordingly, the variant protein conferred a null phenotype. Therefore, no correlation between the catalytic activity and the biological function was found in the new variants either, except in the G285I case (no hydrolytic activity, no function). It should be pointed out that the variants used in this study had no appreciable effect on nucleotide binding (Supplementary Table S1).

**Figure 7.**
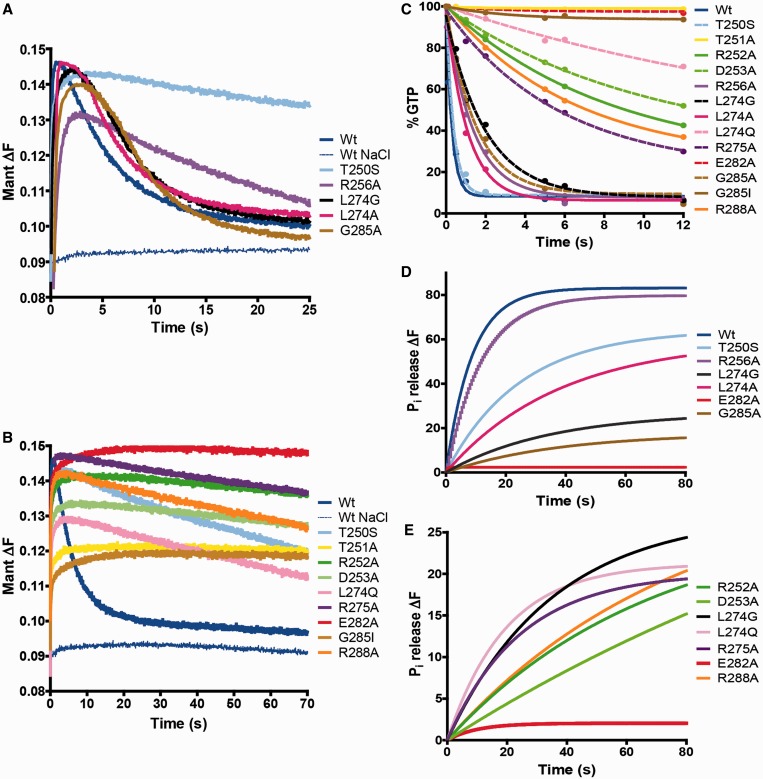
Mutational analysis of the MnmE GTPase cycle. (**A** and **B**) Representative stopped-flow kinetic plots of mGTP binding, G-domain dimerization and G-domain dissociation. Experiments were performed in buffer with KCl, except in a control including the wt MnmE protein where KCl was substituted by NaCl. Fast and slow hydrolase activity variants are shown in panels A and B, respectively. Wt MnmE and variant T250S are used as a reference in both panels. (**C**) GTPase activity of MnmE proteins. Each value is the media from three independent experiments fitted to one-phase exponential decay. (**D** and **E**) Representative time course of P_i_ release from MnmE proteins after GTP hydrolysis fitted to a single-exponential equation. A mix of MnmE and GTP (final concentration, 2 µM) and MDCC-PBP (final concentration, 3.5 μM) was prepared in a fluorescence cuvette, and the P_i_ release was recorded with the spectrophotometer. Fast and slow hydrolase activity variants are shown in panels (D) and (E), respectively, except L274G that has also been included in panel (E) as a reference.

Next, we performed fast kinetic experiments under single-turnover conditions (Figure 7 and Table 5). The rate constants of GTP binding were in the range of 2241–3935 min^−^^1^, with a rate of 3528 min^−^^1^ for the wild-type protein (Table 5). Given that no appreciable differences in the affinity constant (*K*_D_) were found between the wild-type and variant proteins (Supplementary Table S1), we think that the small changes observed in the GTP-binding rate are not responsible for the impaired modification shown by the defective proteins (Table 5).

**Table 5.** Effect of MnmE substitutions on GTPase cycle and tRNA modification status

MnmE protein	GTP binding *k*_1_ (min^−1^)	G-domain dimerization *k*_2_ (min^−1^)	GTP hydrolysis *k_3_* (min^−1^)	G-domain dissociation *k*_4_ (min^−1^)	P_i_ release *k_5_* (min^−1^)	tRNA-modification activity (%)
wt	3528	717	201	12.4 ± 0.6	9.6 ± 1.0	100
Fast hydrolase activity variants						
T250S	3452	356	163	**2.6 ± 0.3**	2.7 ± 0.3	19
R256A	2633	207	44	**5.1 ± 0.8**	5.5 ± 0.6	97
L274G	2761	418	30	11.1 ± 0.6	**1.6 ± 0.1**	0
L274A	3222	378	56	12.0 ± 0.8	**1.5 ± 0.2**	0
G285A	3196	280	42	10.3 ± 0.5	**0.8 ± 0.1**	0
Slow hydrolase activity variants						
T251A	2743	236	**0.01**			0
R252A	3935	755	**6.74**	**1.3 ± 0.1**	**0.8 ± 0.1**	5
D253A	3389	266	**5.71**	**1.7 ± 0.2**	**0.3 ± 0.1**	0
L274Q	3254	222	**2.13**	1.9 ± 0.3	2.4 ± 0.2	19
R275A	3216	218	**12.43**	**1.9 ± 0.2**	2.0 ± 0.2	51
E282A	2241	**104**	**0.02**			0
G285I	2323	**151**	**0.33**			0
R288A	3314	341	**8.22**	**2.3 ± 0.3**	**0.9 ± 0.1**	11

Kinetic constants from single-turnover assays were determined from data as those shown in Figure 7. Results are mean ± SD of at least three independent experiments. When not indicated, standard deviations of rate constants were around ±10%, except for T251A and G285I in which the standard deviation of *k*_3_ was around ±30%. Data on tRNA modification activity of MnmE proteins were taken from Table 4. Bold numbers are used to highlight the implication of the corresponding MnmE substitution in G-domain dimerization, GTP hydrolysis, G-domain dissociation and/or P_i_ release.

Structural analysis has indicated that residues T250, T251, R252, D253, R256 and R288 are directly involved in dimer interface interactions while L274, E282 and G285 are not (13). Our data show that G-domain dimerization occurs in all variants constructed in this study, although at a slightly decreased rate except for R252A (Table 5). The dimerization rate decreased at most 3.5-fold in the majority of variants. Considering that R256A was functionally active (tRNA modification: 97%) yet its G-domain dimerization rate was 3.5-fold lower than that of the wild-type protein, we think that the G-domain dimerization kinetics is not primarily responsible for the modification phenotype of variants. Only E282A and G285I exhibited a G-domain dimerization rate somewhat lower than that of R256A, but they were profoundly affected in the GTP hydrolysis step, which was probably the main cause of their null phenotype.

Analysis of the single-turnover GTPase reaction by quench-flow allowed us to classify the variants into two groups: those exhibiting a GTP hydrolysis rate higher than 30 min^−^^1^ (designated as ‘fast hydrolase activity variants’) and those with a rate lower than 13 min^−1^ (‘slow hydrolase activity variants’) (Figure 7C and Table 5). Variants R256A, L274G, L274A and G285A from the first group had a similar rate (in the range of 30–55 min^−1^), but only R256A (rate constant 44.30 min^−1^) could modify tRNA (Table 5). Protein T250S showed a rate constant close to the wild-type protein (163.20 versus 201.22 min^−1^, respectively) but modified tRNA to a lesser extent than R256A (19 versus 97%, respectively). T250S was particularly affected in G-domain dissociation whereas variants L274G, L274A and G285A were more affected in the P_i_ release step (Table 5 and Figure 7A, C and D). The P_i_ release kinetics depends on kinetics of the previous steps and thus it cannot be significantly faster than the G-domain dissociation kinetics. Given that T250S had a G-domain dissociation and P_i_ release rate constant of 2.6 and 2.7 min^−1^, respectively, we believe that the P_i_ release kinetics is not noticeably affected in this protein. In contrast, L274G, L274A and G285A had G-domain dissociation rates similar to the wild-type protein, but their P_i_ release rates were 6- to 12-fold lower (Table 5 and Figure 3). Therefore, it appears that the P_i_ release defect of these variants is in some way related to the impairment of the MnmE biological function.

The fluorescence amplitude produced by P_i_ release from T250S was lower than that observed in the wild-type protein (Figure 7D), indicating that P_i_ was retained in a certain number of T250S molecules (23% of total molecules retaining P_i_). Amplitude was even lower in L274A and much lower in L274G and G285A (28.2, 67.6 and 78.8%, respectively; Figure 7D). The inhibition constants (*K*_IE_ and *K*_IES_) for P_i_ of T250S and G285A were calculated to be similar to or even slightly higher than those of the wild-type protein, i.e. in none of these cases was the affinity for P_i_ higher than that calculated for the wild-type protein (Figure 5 and Supplementary Figure S6). Therefore, the P_i_ retention appears to be the result of anomalous conformational changes linked with the G-domain dissociation, which would impair the MnmE function. If so, dissociation, rather than hydrolysis, is the driving force for tRNA modification.

Among the ‘slow hydrolase activity variants’ (Table 5 and Figure 7), L274Q and R275A exhibited a tRNA-modification activity identical or even higher than T250S (19 and 51 versus 19%, respectively), despite their GTP hydrolysis rates being ∼76 - and 13-fold slower than that calculated for T250S (Table 5). It should be noted that in the ‘slow hydrolase activity variants’ (and also in mGTPγS hydrolysis by the wild-type protein, see Supplementary Figure S1), the fluorescence observed in the dissociation phase, whose decay was also best fitted to a single-exponential equation, is a composite of fluorescence from both non-hydrolysed mGTP molecules retained by G-domain dimers and mGDP molecules retained by dissociated G-domains. Therefore, in this case, the *k*_4_ value is influenced by the hydrolysis rate. Accordingly, it is possible that a variant with a very low *k*_3_ value but with an efficient G-domain dissociation could exhibit a low *k*_4_ value. This could be the case for L274Q. In this extremely slow variant, G-domain dissociation is not the limiting step, as *k*_3_ and *k*_4_ were similar (Table 5), so G-domain dissociation could not be particularly affected in this protein. In contrast, G-domain dissociation appears to be somewhat impaired in variants R252A, D253A, R275A and R288A, given that their *k*_4_ values were similar to that of L274Q even though their *k*_3_ values were higher. Interestingly, the P_i_ release kinetics does not appear to be affected in R275A and L274Q, as these proteins exhibited a balanced ratio of the dissociation and Pi release rates. In contrast, variants R252A, D253A and R288A were affected in the P_i_ release step, given that their P_i_ release curves did not reach saturation, yet exponential fitting yielded values for the P_i_ release rate (Figure 7E and Table 5). Therefore, a correlation between defects in P_i_ release and the tRNA modification function is also observed in the ‘slow hydrolase activity variants’ (Table 5).

Altogether, these results support the idea that the conformational changes linked with G-domain dissociation, which are affected in variants showing an altered P_i_ release pattern, are responsible for the functional activation of MnmE.

## DISCUSSION

**Figure 8.**
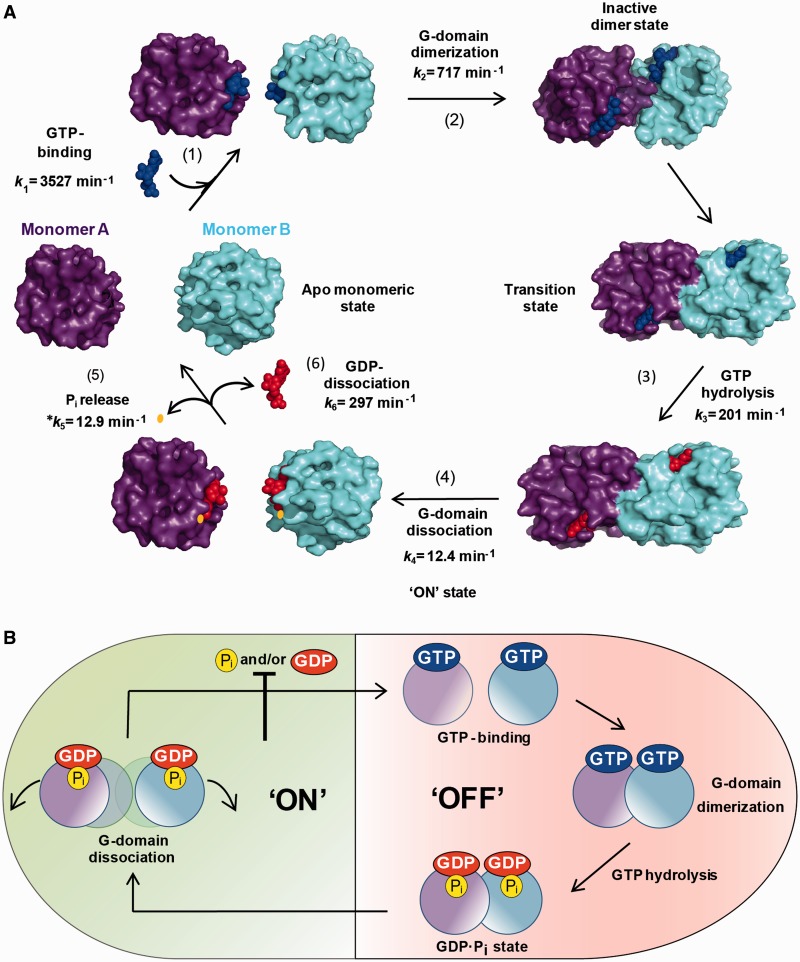
The MnmE GTPase cycle. (**A**) Schematic of conformational changes of the MnmE G-domain during the GTPase cycle. On the left, the G-domains (purple and blue) of a dimeric MnmE protein (not shown) are represented in the apo monomeric state. After GTP binding, a dimeric G-domain is rapidly reorganized to achieve the transition state and promote GTP hydrolysis. Dissociation of G-domain is the rate-limiting step of the GTPase cycle and the driving force for the functional activation of MnmE. P_i_ and GDP are freely released during the G-domain dissociation provided their concentrations in the solution are below the respective *K*_D_. *The P_i_ release rate constant depends on the rates of the previous steps because of the experimental procedure used in this study. Kinetic rate constants were taken from Figure 2. (**B**) The ‘ON’ and ‘OFF’ state of the MnmE G-domain. Unlike other G proteins, the functional activation of MnmE occurs in the post-hydrolysis stage, during G-domain dissociation. GDP and P_i_ work as inhibitors of the GTPase cycle *in vivo*, controlling the GTP loading and GTP hydrolysis, respectively.

We demonstrate that the MnmE GTPase cycle is a sequential process consisting of GTP binding, G-domain dimerization, GTP hydrolysis, G-domain dissociation and release of the reaction products GDP and P_i_ (Figure 8A). Our data establish that G-domain dissociation is the rate-limiting step of the GTPase reaction and the driving force for tRNA modification. By performing kinetic experiments under single-turnover conditions, we show, in contrast to a former report (13), that GTP hydrolysis is faster than the subsequent G-domain dissociation (201 versus 12.4 min^−1^), which appears as the slowest step of the GTPase cycle (Figure 8A). In fact, the dissociation rate (12.4 min^−1^) is similar to *k*_cat_ (11.4 min^−^^1^), a strong indication that the physical step of G-domain dissociation limits the final rate of the hydrolysis reaction. Moreover, G-domain dissociation controls the release of GDP and P_i_. The conformation of the G-domain dimer in the transition state (Figure 4) prevents the release of the reaction products, which may occur instantaneously when the dimer is undone and the switches are disengaged. It is worth to noting that the homodimerization of Toc33, a GTPase involved in protein translocation into chloroplasts, has been reported to reduce the rate of GDP release by capturing the nucleotide in a cage at the dimer interface (38). Thus, Toc33 provides another example of how the nucleotide release is inhibited by dimerization.

In the stopped-flow experiments, we detected a short lag phase (about 0.6 s) that fits well with the run-time of GTP hydrolysis (Figure 3B). This outcome strongly suggests that GTP hydrolysis and the formation of a GDP•P_i_ intermediate take place in the lag phase and that they do not entail an instantaneous conformational change at the G-domain active centre. GTP hydrolysis in G proteins leads to conformational changes in switches I and II, but the homodimeric conformation of the MnmE G-domains, by conferring stability to the switches, probably slows down the conformational rearrangements following GTP hydrolysis. In this way, G-domain dissociation is delayed and becomes slower than the hydrolysis reaction.

Considering that GTP hydrolysis is essential for tRNA modification, the molecular rearrangements leading to the functional activation of MnmE could occur during dimerization of the G-domains that is associated with stabilization of the transition state, as previously hypothesized (16,18), or during G-domain dissociation where the relaxation of switches I and II should take place. Our mutational analysis supports the latter hypothesis. This analysis indicates that GTP hydrolysis and G-domain dissociation can be uncoupled, and that variants showing a high hydrolysis rate, but altered post-hydrolysis steps, are defective in tRNA modification (Table 5 and Figure 7D). Dissociation is affected in T250S, which exhibits a 5-fold slower dissociation rate than the wild-type protein, yet hydrolyses GTP almost at the wild-type rate. Note that residue T250 belongs to the G2 motif where it is adjacent to the canonical threonine (T251 in MnmE; Figure 6A) involved in the so-called loaded-spring mechanism. This mechanism is responsible for the relaxation of switches I and II after GTP hydrolysis, and it is believed to be universal (1,39). In the transition-state structure of the MnmE G-domain (Figure 6D), T250 contacts the γ phosphate of GTP through its main chain and residues T245 and I247, which are located in the K-loop of switch I and participate in potassium coordination, through its side chain (13). Any significant alteration of these interactions should have a drastic effect on GTP hydrolysis. Since this was not the case in T250S (Table 5), we assume that the conformation of its switch I in the transition state is similar to that of the wild-type protein. Accordingly, the functional impairment of T250S (which exhibits 19% of the tRNA modification level in relation to the wild-type protein) should be due to events occurring after GTP hydrolysis and associated with the G-domain dissociation step. Note, however, that R256A, which displays a wild-type tRNA modification function, also exhibited a slow dissociation rate, although not as slow as T250S (Table 5). In any case, the R256A features prompt us to be cautious about the possibility that the dissociation kinetic is the main cause of the functional impairment of T250S.

Our data also reveal that the dissociation and P_i_ release steps can be uncoupled given that L274G, L274A and G285A exhibit 6- to 12-fold slower P_i_ release rates than the wild-type protein, yet their dissociation rates achieve the wild-type level (Table 5). These variants cannot modify tRNA even though they hydrolyse GTP at the same rate as protein R256A, which has a wild-type phenotype. Notably, P_i_ is retained by a significant fraction of molecules in T250S as well as in L274A, L274G and G285A (Figure 7D), with this feature being the differentiating and specific characteristic of the four variants. As we found that the affinity of T250S and G285A for P_i_ is in the wild-type protein range, we conclude that the P_i_ retention by these variants is the result of anomalous molecular rearrangements, which would be the cause of their defective capability to modify tRNA. Altogether, these results support the idea that G-domain dissociation is responsible for the ‘ON’ state of MnmE, which introduces a new paradigm in the cycling mechanism used by GTPases to regulate cellular functions (Figure 8B).

Residue T250 appears to play a crucial role in the unwinding of the G-domain dimer from its strategic position within the GTP-binding site (see above and Figure 6D). We speculate that T250 senses GTP hydrolysis and transmits a signal to both residues T245 and I247 of the K-loop, thus promoting disengagement of potassium and switch I, as well as to nearby residues A248 and R252, thus breaking up their interactions with residues R275 and D279 of switch II of the same protomer. The breaking of interactions A248−R275 and R252−D279 would lead to disengagement of switches I and II, facilitating disassembly of the dimer and, at the same time, promoting the opening of the cage where GDP and P_i_ are located on each protomer. The change of T250 to Ser could yield stronger interactions with residues T245 and I247, hindering their breaking and thus slowing down the dissociation rate. More importantly, substitution T250S (as well as L274A, L274G and G285A) would alter the conformational changes of switches I and II associated with their disengagement, promoting the retention of P_i_. In contrast, substitution of the non-conserved R256 residue to Ala, even though affecting the dissociation rate, appears not to significantly influence such post-hydrolysis rearrangements, given that the variant protein confers an almost wild-type phenotype and releases P_i_ as fast as the dimer dissociation allows it (Table 5).

A disengagement of switch I after GTP hydrolysis has also been suggested to promote G-domain dissociation in dynamin, which, similarly to MnmE, is a GAD that requires GTP hydrolysis for functional activation (40,41). The relationships between GTP hydrolysis and the membrane-remodeling events promoted by dynamin-like proteins remain unclear (40–42). Our present work offers a new perspective of how GADs may use the GTPase cycle to drive their biological activity.

Several members of the TRAFAC class (translation factors) of GTPases contain a TT sequence in their G2 motif (e.g., Obg, DRG, ERA, EngAb, YqeH and Toc159) (43,44). Thus, it is possible that the threonine adjacent to the catalytic one could play a linking role between the GTPase cycle and the functionally relevant activity of these proteins, as herein shown for MnmE.

Given that modification of tRNA by the MnmEG complex requires GTP hydrolysis, it appears reasonable that the MnmE GTPase cycle and the MnmEG function are coupled and that the GTPase activity of MnmE is regulated in some way to avoid futile GTP hydrolysis. Our data demonstrate that the GTPase cycle is negatively controlled by the reaction products GDP and P_i_. GDP acts as an inhibitor of GTP binding whereas P_i_ has a major effect on stabilization of the transition state. While GDP acts like a purely competitive inhibitor, P_i_ acts as a mixed inhibitor. As far as we know, this is the first time that P_i_ is identified as a mixed inhibitor of GTPase activity. P_i_ could bind to a site in the MnmE catalytic centre without affecting GTP binding but hindering the formation of the transition state. In MnmE (and human dynamin), the correct positioning of a water molecule for a nucleophilic attack on the γ-phosphate is performed in an indirect way by means of a second water molecule (13,41). In the active site of each MnmE G-domain, the attacking water is stabilized by residue G249 from the G2 motif and by another water molecule, which in turn is hydrogen-bonded by G273 from the G3 motif and E282 (13). It is possible that P_i_ disturbs the stabilization of the transition state by interfering with the positioning of the catalytic or the bridging water.

Our data indicate that the GDP and P_i_ release from MnmE occurs instantaneously under non-inhibitory concentrations of both products. If so, neither GDP release nor P_i_ release play a direct role in the mechanism driving tRNA modification. Thus, MnmE differs from some translation factors in which the GDP•P_i_ form and the dissociation of P_i_ appear to play an important mechanistic role (45–48).

From the IC_50_ values, we conclude that at GDP and P_i_ physiological steady-state concentrations, the MnmE GTPase cycle is inhibited, and suggest that a conformational change of MnmE could be required to remove the inhibitors, allowing GTP accommodation and leading to the beginning of a new round (Figure 8B). This change could be triggered by the binding of tRNA to the MnmEG complex (Supplementary Figure S7). The MnmE G-domain is relatively far from the active centre of the MnmEG complex. However, previous findings have indicated that interactions of MnmE with MnmG on a site remote from the MnmE G-domain co-stimulate GTP hydrolysis (18,49). Therefore, it is possible that tRNA binding to the MnmEG active centre might modulate the GTPase cycle. In fact, activity of many translation-associated GTPases has been found to be regulated by RNA binding (4).

In conclusion, our data define the tRNA-modifying protein MnmE as a paradigmatic ‘non-Ras’ GTPase in which the cycle is regulated by the hydrolysis products GDP and P_i_, and the ‘ON’ state is achieved in a post-hydrolysis step. Thus, our work expands conventional views regarding the action mechanism and regulation of GTPases, and provides new insights on the role of GTP hydrolysis in MnmE-dependent tRNA modification.

## SUPPLEMENTARY DATA

Supplementary Data are available at NAR Online: Supplementary Tables 1 and 2, Supplementary Figures 1–7, Supplementary Methods and Supplementary References [10,12,13,19,20,36,50].

## FUNDING

Spanish Ministry of Economy and Competitiveness [BFU2007-66509 and BFU2010-19737 to M.-E.A.]; Generalitat Valenciana [ACOMP/2012/065 to M.-E.A.]; [PROMETEO/2012/061 to J. Bravo]; postdoctoral fellowship from Bancaja (to S.P.). Funding for open access charge: [BFU2010-19737 to M.-E.A.].

*Conflict of interest statement.* None declared.

## Supplementary Material

Supplementary Data
