# Supplementary material for: The tRNA-modifying function of MnmE is controlled by post-hydrolysis steps of its GTPase cycle
Source: Nucleic Acids Res. 2013 Apr 27;41(12):6190–208. doi: 10.1093/nar/gkt320 (PMC3695501; doi:10.1093/nar/gkt320)
Supplement: Supplementary Data [file supp_41_12_6190__index.html]

The tRNA-modifying function of MnmE is controlled by post-hydrolysis steps of its GTPase cycle — The tRNA-modifying function of MnmE is controlled by post-hydrolysis steps of its GTPase cycle — Supplementary Data 

# The tRNA-modifying function of MnmE is controlled by post-hydrolysis steps of its GTPase cycle

## Supplementary Data

files

**Files in this Data Supplement:**

- Supplementary Data - pdf file
