# Supplementary material for: The tRNA-modifying function of MnmE is controlled by post-hydrolysis steps of its GTPase cycle
Source: Nucleic Acids Res. 2013 Apr 27;41(12):6190–208. doi: 10.1093/nar/gkt320 (PMC3695501; doi:10.1093/nar/gkt320)
Supplement: Supplementary Data [file supp_gkt320_nar-03293-r-2012-File010.pdf]

SUPPLEMENTARY DATA

Post-Hydrolysis Steps Control Functional Activation of the tRNA modification GTPase MnmE

Silvia Prado, Magda Villarroya, Milagros Medina and M.-Eugenia Armengod

[1] RNA Modification and Mitochondrial Diseases Laboratory, Centro de Investigación Príncipe Felipe, 46012-Valencia, Spain
[2] Departamento de Bioquímica, Biología Molecular y Celular. Instituto de Biocomputación y Física de Sistemas Complejos (BIFI). Universidad de Zaragoza, 50009-Zaragoza, Spain.
[3] CIBERER (node U721), Spain

Corresponding author:
M.-Eugenia Armengod
RNA Modification and Mitochondrial Diseases Laboratory
Centro de Investigación Príncipe Felipe
Calle de Eduardo Primo Yúfera, 3
46012-Valencia, Spain
E-mail: armengod@cipf.es
Phone: 34-963289681 (2006#)
Fax: 34-963289701

Inventory of Supplementary Data:

Supplementary Material and Methods

Supplementary Figure Legends

Supplementary Tables

Supplementary References

Supplementary Figures

Supplementary Material and Methods

Strain construction, DNA manipulations, and general protein techniques. Genetic techniques for the construction of strains were performed as described previously (1). The substitution of the *mnmE* wild-type allele by mutant alleles on the *E. coli* chromosome was initially performed by homologous recombination between linearized plasmids (derived from pIC914) containing the *mnmE* mutant alleles and the chromosome of strain IC3647. P1 stocks grown on the IC3647 derivatives thus obtained were then used to transfer the *mnmE* mutant alleles to the desired strains. For DNA manipulations, standard procedures were followed. Derivatives from plasmids pIC684, pIC758, pIC1325, and pIC1335 were obtained by site-directed oligonucleotide mutagenesis with appropriated PCR primers. All constructs were verified by DNA sequencing.

Expression and purification of GST-MnmE proteins (wild-type and variants) and the GST−G-domain of MnmE were performed in *E. coli* strain DEV16 as described (2). Overproduction of His−ΔN-MnmE was done in strain BL21DE3 growing overnight at 20ºC in rich medium with 0.5 mM isopropyl-1-thio-β-D-galactopyranoside. Cells were harvested by centrifugation and resuspended in lysis buffer: 50 mM Tris pH 7.5, 300 mM NaCl, 5 mM $MgCl_2$, 5 mM β-mercaptoethanol, 20 mM imidazole, and 0.15 mM protease inhibitor phenylmethylsulphonyl fluoride. After sonication, samples were centrifuged at 35,000 ×*g* for 30 min and the supernatant applied to a Ni-NTA column. The column was washed with lysis buffer and the protein eluted with the same buffer containing 250 mM imidazole. Fractions were concentrated and purified by gel filtration on a Superdex™75HR10/30 (50 mM Tris pH 7.5, 150 mM KCl, and 5 mM $MgCl_2$). Preparation of nucleotide-free proteins was performed as previously described (3) except that MnmE constructs were finally purified by gel filtration and the resulting aliquots were concentrated in buffer A (50 mM Tris pH 7.5, 150 mM KCl, and 5 mM $MgCl_2$).

Supplementary Figure Legends

Figure S1. Comparision of the MnmE GTPase cycle using mGTP and mGTPγS as a substrate. (A, B) Stopped-flow kinetic plots of mGTP binding and G-domain dimerization during the first 0.5 s, in A, and G-domain dissociation during 200 s, in B. (C, D) Stopped-flow traces of mGTPγS binding and G-domain dimerization during the first 0.5 s, in C, and G-domain dissociation during 200 s, in D.

Figure S2. GDP-GTP exchange kinetics for MnmE. Representative traces of mGDP dissociation from MnmE determined by stopped-flow FRET. MnmE was mixed with mGTP and FRET from tryptophans of MnmE to the mant group was followed in real time after addition of unlabeled competitor GTP at the indicated concentrations.

Figure S3. Inhibition by GDP and P_i of the MnmE GTPase cycle. A solution of MnmE (5 μM) containing the indicated concentrations of GDP (panel A) or P_i (panel B) was rapidly mixed in a 1:1 proportion with mGTP (5 μM) in a stopped-flow apparatus and the mant fluorescence emission was followed in real time. The fluorescence decay expected if inhibition by P_i at 10 mM were 75% is shown in pale blue in panel B. Only a representative trace of each experiment is shown, but each experiment was repeated three times. All experiments were performed in the presence of KCl except a control where KCl was substituted by NaCl (gray line).

Figure S4. Fast kinetic analysis of the MnmE GTPase cycle in presence of high ionic strength. (A) A stopped-flow representative trace of mGTP binding and G-domain dimerization during the first 0.5 s. (**B**) G-domain dissociation representative trace followed by stopped-flow during 10 s.

Figure S5. High performance liquid chromatography analysis of the tRNA modification activity displayed by MnmE variants. Representative chromatograms of tRNA hydrolysates prepared from wild-type and *mnmE* mutants. The nucleosides were monitored at 314 nm to maximize the detection of thiolated nucleosides. mnm^5s^2U (the final product of the MnmE pathway), s^2U (a partial modification of the wobble uridine, U34, if the MnmE pathway is impaired) and s^4U (a nucleoside independent of the MnmE pathway and used herein as a reference) were identified by comparing UV spectra with published spectra (4) and appropriate controls. *AU, absorbance* units.

Figure S6. P_i inhibition of the MnmE variants T250S and G285A. A solution of the MnmE variant (5 μM) containing different P_i concentrations (0, 0.5, 2, 5, or 10 mM) was mixed in a 1:1 proportion with mGTP (at 5, 10 or 15 μM) in the stopped-flow instrument and the G-domain dissociation kinetics was determined by the fluorescence change over time and fitting of the data to a single exponential. Values are the average of a minimum of three independent experiments. Standard deviations were around ±10%. Dixon plot of T250S (**A**) and G285A (**B**). [GTP]/V_o against [P_i] representation of T250S (**C**) and G285A (**D**). The intersection point of lines in the Dixon plot provides the inhibition constant (K_{IE}) of the enzyme for the inhibitor, while the intersection point in the [GTP]/V_o *vs* [P_i] representation provides the inhibition constant of the enzyme-substrate complex (K_{IES}) for the inhibitor.

Figure S7. Active conformational state of the MnmEG complex. Proposed model for the functional activation of the MnmEG complex during the GTPase cycle of MnmE. The two monomers of the dimeric MnmE and MnmG proteins are shown in purple/blue and in pale-gray/yellow, respectively. GTP-binding, G-domain dimerization and GTP-hydrolysis are non-functional states (‘OFF’ states) of MnmE whereas G-domain dissociation represents the ‘ON’ state because it promotes the conformational changes (black arrows) that are required for the MnmE biological function, i.e., the tRNA modification. The G-domain dissociation triggers structural rearrangements in the complex which are transmitted from the G-domain of MnmE to MnmG promoting the assembly of the MnmEG catalytic center. This involves the approaching of the THF-binding site of MnmE and the FAD-binding site of MnmG. Release of modified tRNA disassembles the MnmEG active center. The GTPase reaction products, GDP and P_i, work as negative regulators of the GTPase cycle and, therefore, of the MnmEG function. Conformational changes promoted by the binding of unmodified tRNA trigger the release of GDP and P_i while allowing the entry of GTP and the start of a new cycle.

Supplementary Tables

Table S1. Affinity of wt and variant MnmE proteins for mGTPγS and mGTP.

	K_D (μM)	
	**mGTPγS**	**mGTP**
**wt**	1.64 ± 0.02	0.60 ± 0.03
**T250S**	1.34 ± 0.12	nd
**T251A**	1.58 ± 0.03	nd
**R252A**	1.97 ± 0.27	nd
**D253A**	2.35 ± 0.13	nd
**R256A**	1.49 ± 0.12	0.59 ± 0.04
**L274G**	1.29 ± 0.06	nd
**L274A**	1.22 ± 0.04	nd
**L274Q**	1.44 ± 0.09	nd
**R275A**	1.33 ± 0.09	nd
**E282A**	1.14 ± 0.04	0.54 ± 0.04
**G285A**	2.52 ± 0.11	nd
**G285I**	1.87 ± 0.20	nd
**R288A**	1.27 ± 0.20	nd

K_D for mGTPγS was determined under equilibrium conditions. K_D for mGTP was derived from kinetic data (pre-equilibrium binding conditions). nd, not done.

Table S2. *E. coli* strains and plasmids used in this study.

Strain or plasmid	Description	Origin and/or reference
***E.coli* strain**		
**BL21(DE3)**		Novagen
**DEV16**	*thi-1 rel-1 spoT1 lacZ105*$_{UAG}$ *mnmE192*$_{UAG}$ [MnmE Q192X, Val^R]	(5)
**MG1655**		D. Touati
**IC3647**	*recBC sbcBC* (λIC718)	(6)
**IC4639**	DEV16 *mnmE*$^+$ *bgl*	(7)
**IC4770**	DEV16 mnmE$^+$ kan^R*	(6)
**IC4848**	DEV16 mnmE*::kan*	(6)
**IC4864**	IC4639 *mnmE*R252A kan^R*	(6)
**IC4865**	IC4639 *mnmE*R275A kan^R*	(6)
**IC5357**	MG1655 mnmE$^+$ kan^R*	(6)
**IC5358**	MG1655 *mnmE::kan*	(6)
**IC5360**	MG1655 *mnmE*R288A kan^R*	(6)
**IC5361**	MG1655 *mnmE*T250S kan^R*	(6)
**IC5363**	MG1655 *mnmE*E282A kan^R*	This work
**IC5423**	MG1655 *mnmE*L274A kan^R*	This work
**IC5365**	MG1655 *mnmE*L274Q kan^R*	This work
**IC4907**	IC4639 *mnmE*T251A kan^R*	(6)
**IC4956**	IC4639 *mnmE*D253A kan^R*	(6)
**IC5019**	IC4639 *mnmE*R288A kan^R*	(6)
**IC5058**	IC4639 *mnmE*T250S kan^R*	(6)
**IC5113**	IC4639 *mnmE*R256A kan^R*	(6)
**IC5366**	IC4639 *mnmE*E282A kan^R*	This work
**IC6031**	BL21(DE3) *mnmE::kan*	This work
**IC6597**	MG1655 *mnmE::kan* containing pIC1657	This work
**IC6598**	BL21(DE3) *mnmE::kan* containing pIC1658	This work
**IC6599**	BL21(DE3) *mnmE::kan* containing pIC1659	This work
**Plasmids**		
**pIC684**	GST fusion of MnmE (cloned in pGEX-2T)	(2)
**pIC758**	GST fusion of MnmE G-domain (cloned in pGEX-2T)	(2)
**pIC914**	Plasmid containing the *mnmE-tnaA* region*	(6)
**pIC938**	pIC684 derivative containing *mnmE*R275A	(6)
**pIC939**	pIC684 derivative containing *mnmE*R252A	(6)
**pIC1003**	pIC684 derivative containing *mnmE*T251A	(6)
**pIC1013**	pIC684 derivative containing *mnmE*D253A	(6)
**pIC1050**	pIC684 derivative containing *mnmE*R288A	(6)
**pIC1059**	pIC684 derivative containing *mnmET*250S	(6)
**pIC1072**	pIC684 derivative containing *mnmE*R256A	(6)
**pIC1090**	pIC684 derivative containing *mnmE*L274A	This work
**pIC1325**	His fusion of MnmE (cloned in pET15b)	This work
**pIC1335**	His fusion of ΔN-MnmE (cloned in pET15b)	This work
**pIC1657**	pIC684 derivative containing *mnmE*G285A	This work
**pIC1658**	pIC1325 derivative containing *mnmE*G285I	This work
**pIC1659**	pIC1325 derivative containing *mnmE*L274G	This work

* Kan^R determinant is inserted into *tnaA* (gene next to *mnmE*).

Supplementary References

1. Miller, J.H. (1992) A Short Course in Bacterial Genetics *Cold Spring Harbor*.
2. Cabedo, H., Macian, F., Villarroya, M., Escudero, J.C., Martinez-Vicente, M., Knecht, E. and Armengod, M.E. (1999) The Escherichia coli trmE (mnmE) gene, involved in tRNA modification, codes for an evolutionarily conserved GTPase with unusual biochemical properties. *EMBO J*, **18**, 7063-7076.
3. Scrima, A. and Wittinghofer, A. (2006) Dimerisation-dependent GTPase reaction of MnmE: how potassium acts as GTPase-activating element. *EMBO J*, **25**, 2940-2951.
4. Gehrke, C.W. and Kuo, K.C. (1989) Ribonucleoside analysis by reversed-phase high-performance liquid chromatography. *J Chromatogr*, **471**, 3-36.
5. Elseviers, D., Petrullo, L.A. and Gallagher, P.J. (1984) Novel E. coli mutants deficient in biosynthesis of 5-methylaminomethyl-2-thiouridine. *Nucleic Acids Res*, **12**, 3521-3534.
6. Martinez-Vicente, M., Yim, L., Villarroya, M., Mellado, M., Perez-Paya, E., Bjork, G.R. and Armengod, M.E. (2005) Effects of mutagenesis in the switch I region and conserved arginines of Escherichia coli MnmE protein, a GTPase involved in tRNA modification. *J Biol Chem*, **280**, 30660-30670.
7. Yim, L., Moukadiri, I., Bjork, G.R. and Armengod, M.E. (2006) Further insights into the tRNA modification process controlled by proteins MnmE and GidA of Escherichia coli. *Nucleic Acids Res*, **34**, 5892-5905.

Figure S1

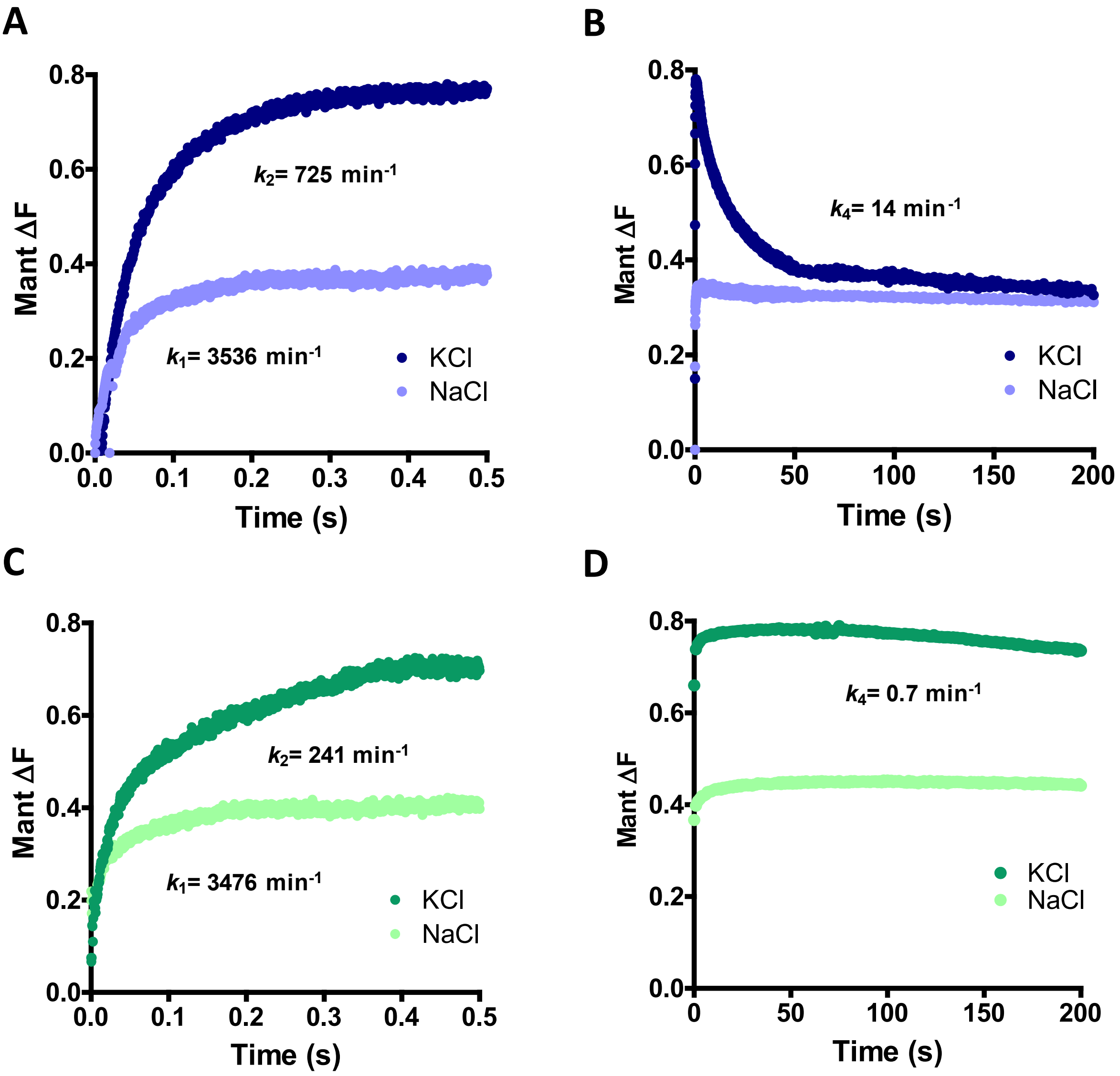
A
B
C
D
Mant ΔF
Time (s)
k2= 725 min-1
k1= 3536 min-1
k4= 14 min-1
k2= 241 min-1
k1= 3476 min-1
k4= 0.7 min-1
KCl
NaCl
0.0
0.2
0.4
0.6
0.8
0.1
0.3
0.5
0
50
100
150
200

Figure S2

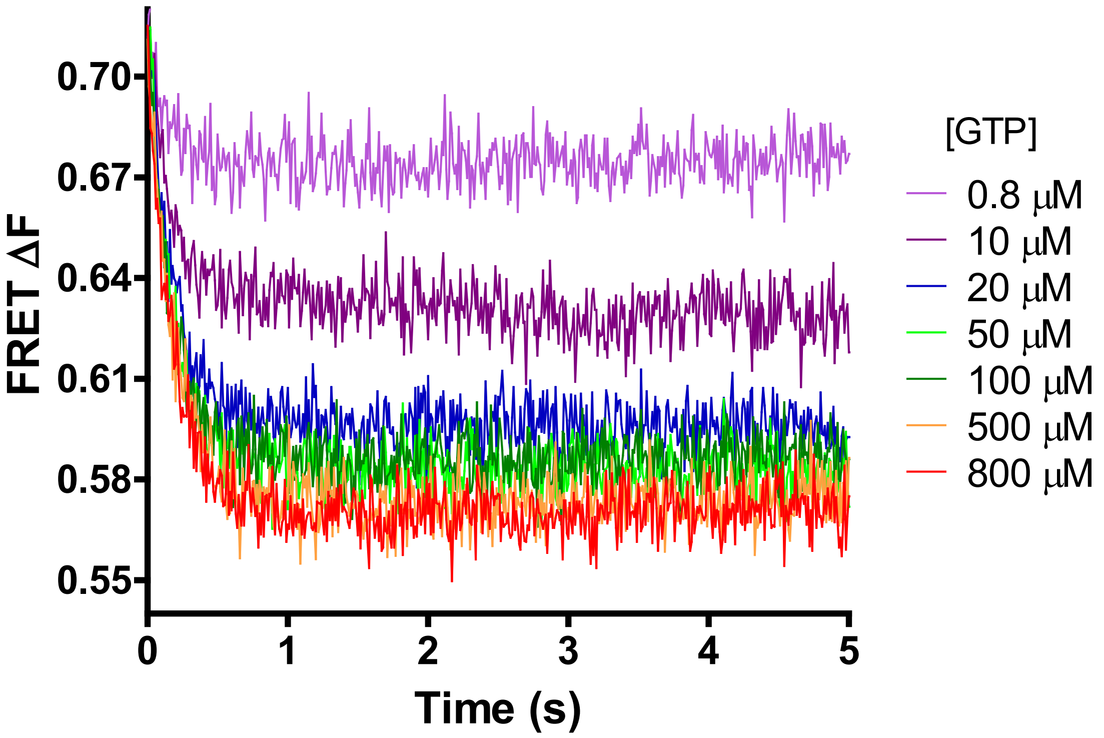

FRET ΔF
0.70
0.67
0.64
0.61
0.58
0.55
0
1
2
3
4
5
Time (s)
[GTP]
0.8 μM
10 μM
20 μM
50 μM
100 μM
500 μM
800 μM

Figure S3

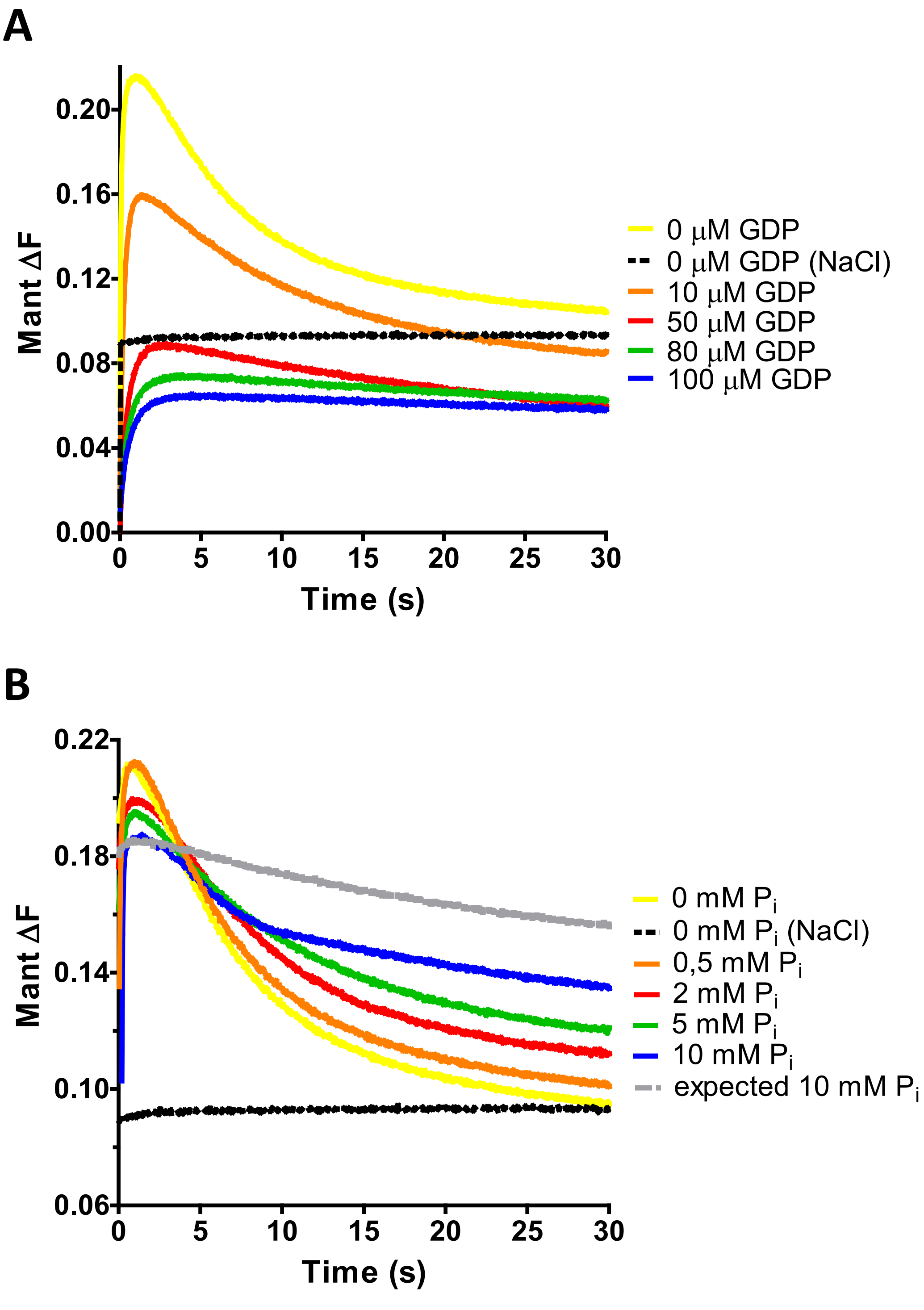
A
Mant ΔF
0.00
0.04
0.08
0.12
0.16
0.20
0
5
10
15
20
25
30
Time (s)
0 μM GDP
0 μM GDP (NaCl)
10 μM GDP
50 μM GDP
80 μM GDP
100 μM GDP
B
Mant ΔF
0.06
0.10
0.14
0.18
0.22
0
5
10
15
20
25
30
Time (s)
0 mM P_i
0 mM P_i (NaCl)
0,5 mM P_i
2 mM P_i
5 mM P_i
10 mM P_i
expected 10 mM P_i

Figure S4

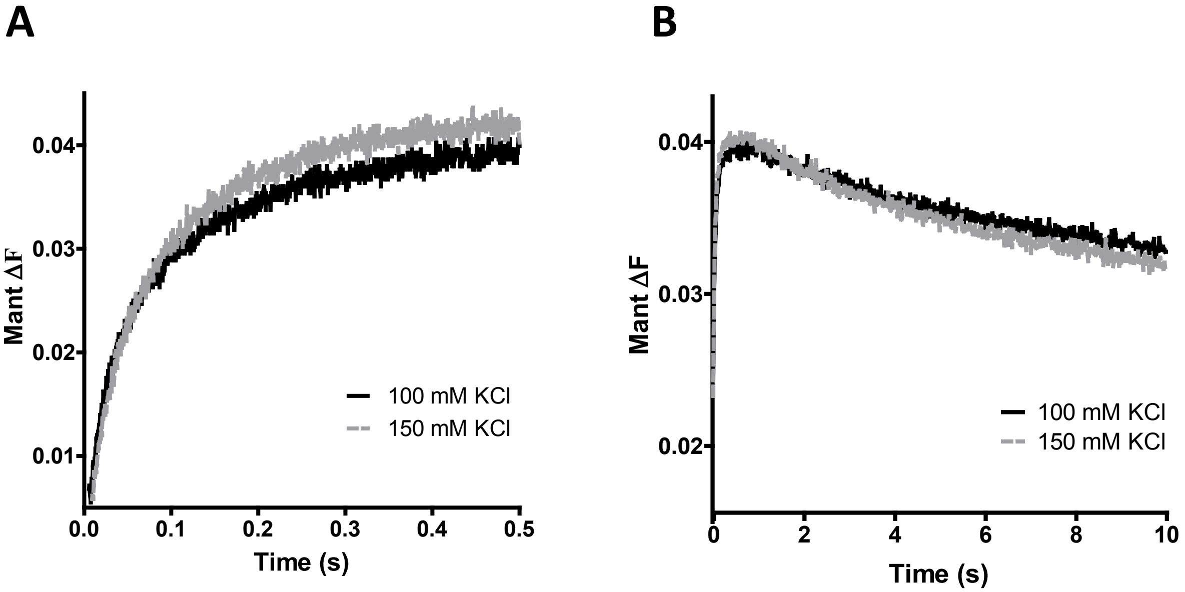
A
Mant ΔF
0.04
0.03
0.02
0.01
0.0
0.1
0.2
0.3
0.4
0.5
Time (s)
100 mM KCl
150 mM KCl
B
Mant ΔF
0.04
0.03
0.02
0
2
4
6
8
10
Time (s)
100 mM KCl
150 mM KCl

Figure S5

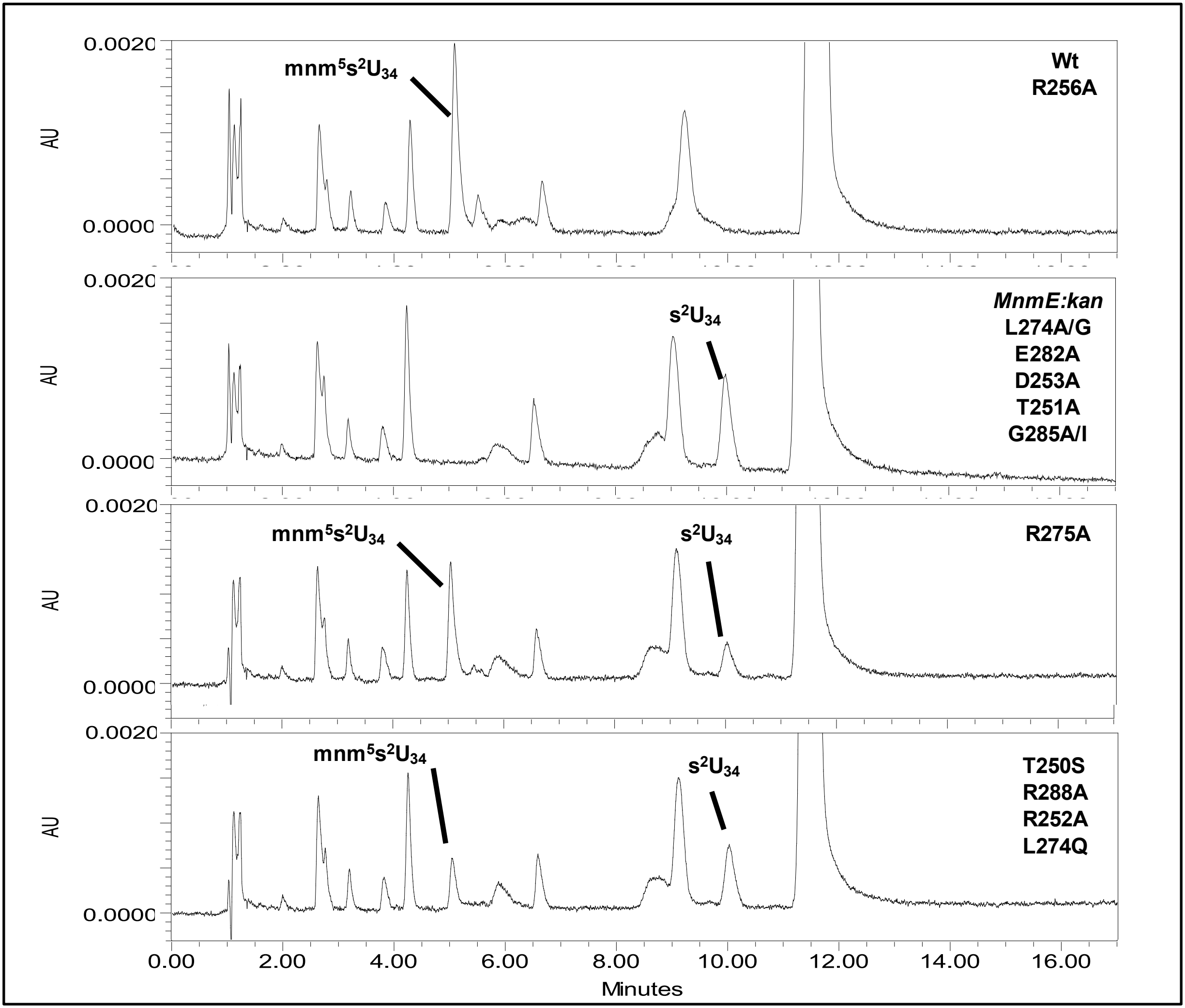

0.0020
0.0000
AU
mnm⁵s²U34
Wt
R256A
MnmE:kan
L274A/G
E282A
D253A
T251A
G285A/I
s²U34
R275A
T250S
R288A
R252A
L274Q
0.00
2.00
4.00
6.00
8.00
10.00
12.00
14.00
16.00
Minutes

Figure S6

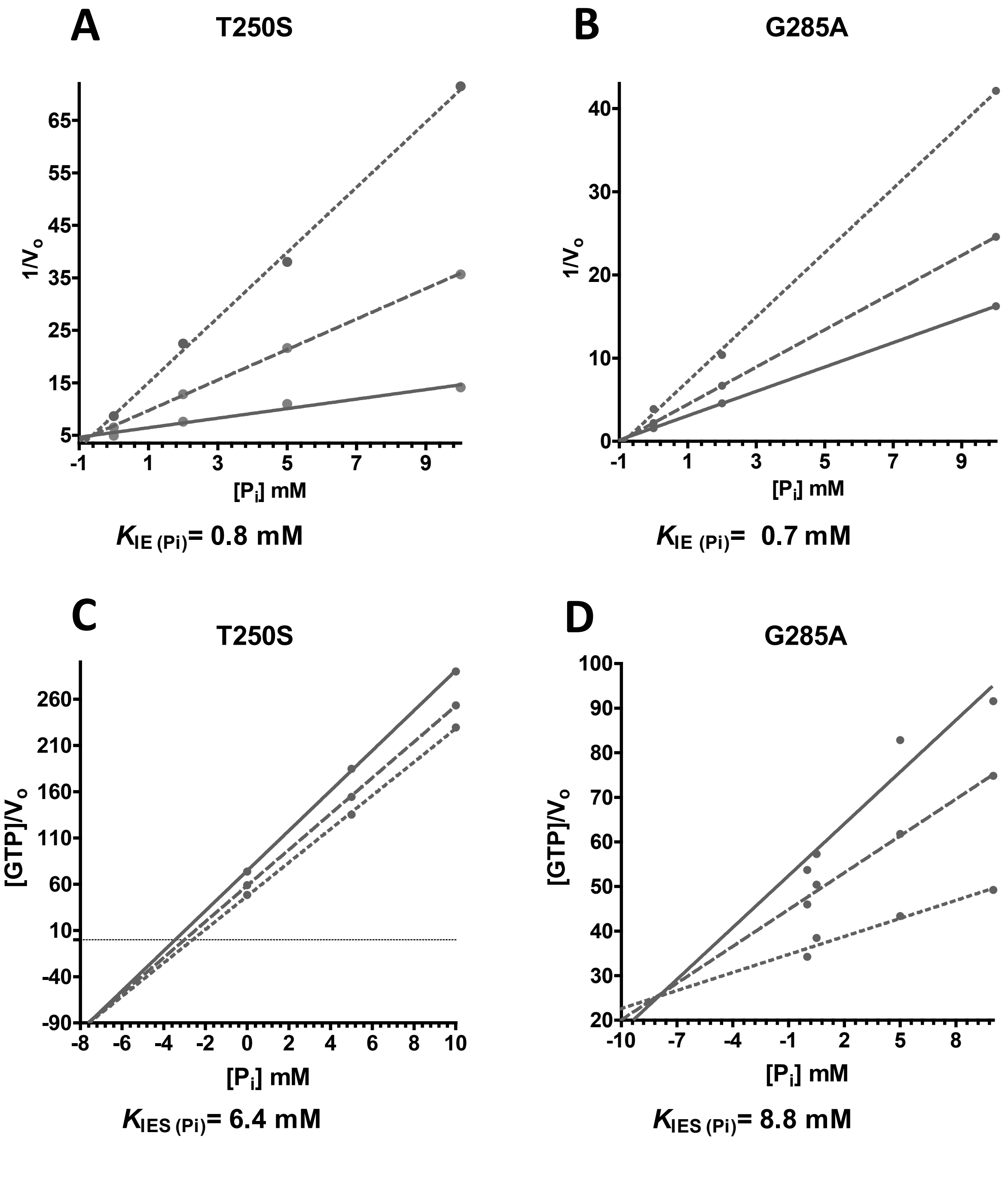
A
T250S
1/Vo
[Pi] mM
$K_{IE\ (Pi)}$= 0.8 mM
B
G285A
1/Vo
[Pi] mM
$K_{IE\ (Pi)}$= 0.7 mM
C
T250S
[GTP]/Vo
[Pi] mM
$K_{IES\ (Pi)}$= 6.4 mM
D
G285A
[GTP]/Vo
[Pi] mM
$K_{IES\ (Pi)}$= 8.8 mM

Figure S7

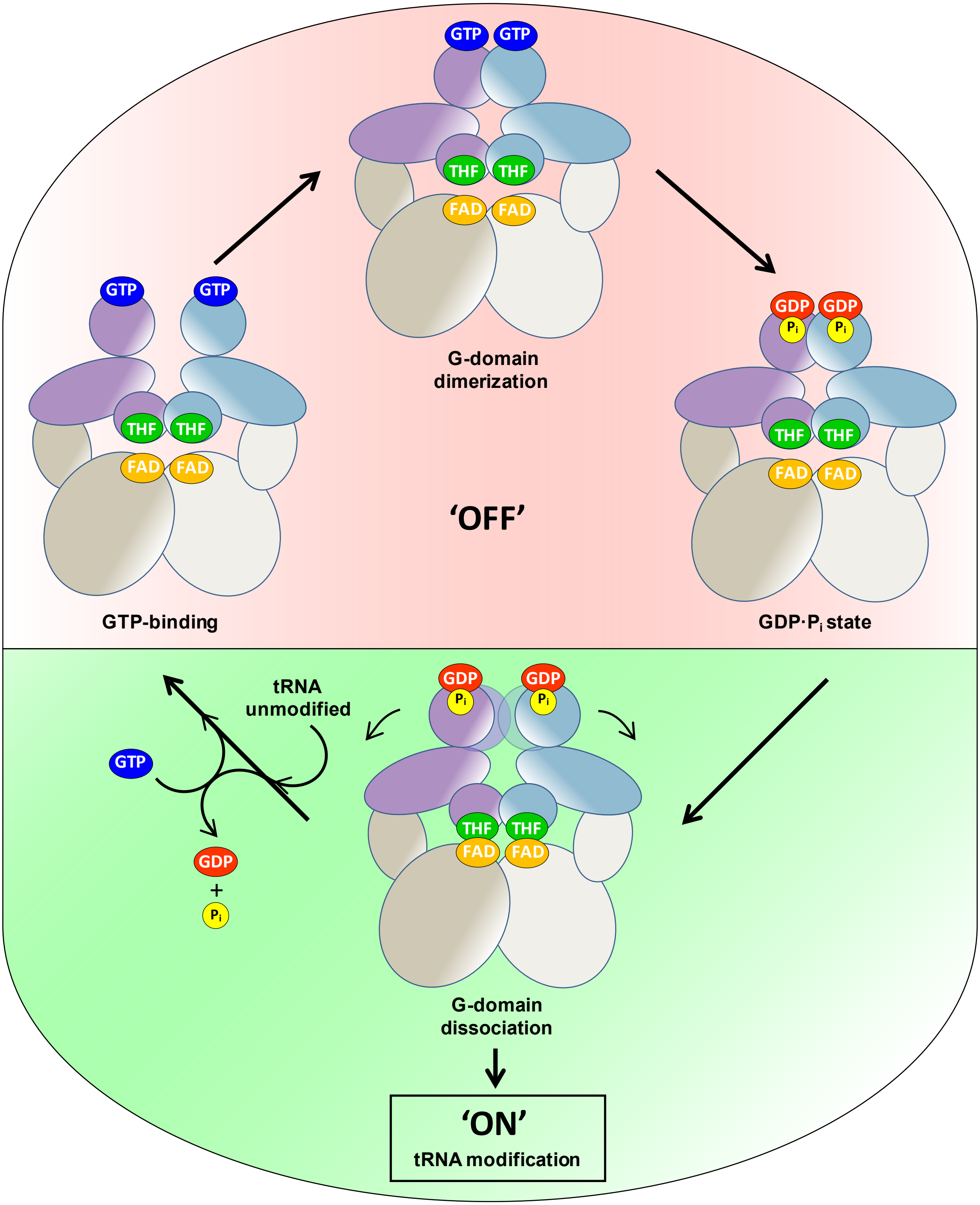

GTP
GTP
THF
THF
FAD
FAD
G-domain dimerization
GTP
GTP
THF
THF
FAD
FAD
GTP-binding
'OFF'
GDP
GDP
Pi
Pi
THF
THF
FAD
FAD
GDP·Pi state
tRNA unmodified
GTP
GDP
+
Pi
GDP
GDP
Pi
Pi
THF
THF
FAD
FAD
G-domain dissociation
'ON'
tRNA modification
